# Blood Pressure and Mortality in Mexico City: A Mendelian Randomization Study

**DOI:** 10.1161/HYPERTENSIONAHA.125.25348

**Published:** 2025-09-17

**Authors:** Michael Turner, Pablo Kuri-Morales, Jesús Alegre-Díaz, Paulina Baca, Jose Adrián Garcilazo-Ávila, Carlos González-Carballo, Raul Ramirez-Reyes, Fernando Rivas, Diego Aguilar-Ramírez, Fiona Bragg, Louisa Gnatiuc Friedrichs, William G. Herrington, Michael Hill, Eirini Trichia, Alejandra Vergara-Lope, Rachel Wade, Doreen Zhu, Rory Collins, Richard Peto, Jaime Berumen, Natalie Staplin, Jason Torres, Richard Haynes, Jonathan R. Emberson, Roberto Tapia-Conyer

**Affiliations:** Clinical Trial Service Unit and Epidemiological Studies Unit, Nuffield Department of Population Health (NDPH), University of Oxford, United Kingdom (M.T., D.A.-R., F.B., L.G.F., W.G.H., M.H., E.T., A.V.-L., R.W., D.Z., R.C., R.P., N.S., J.T., R.H., J.R.E.).; Instituto Tecnológico y de Estudios Superiores de Monterrey, Mexico (P.K.-M.).; Faculty of Medicine, UNAM, Mexico City, Mexico (P.K.-M., R.T.-C.).; Experimental Research Unit, Faculty of Medicine, National Autonomous University of Mexico (UNAM), Mexico City (J.A.-D., P.B., J.A.G.-A., C.G.-C., R.R.-R., F.R., J.B.).; Health Data Research UK Oxford, University of Oxford, United Kingdom (F.B.).

**Keywords:** blood pressure, cardiovascular diseases, humans, kidney, Mexico, prospective studies

## Abstract

**BACKGROUND:**

Observational studies relating blood pressure in middle age to mortality may underestimate lifelong effects. Mendelian randomization can reduce the impact of confounding and reverse causality and may better estimate lifelong effects of blood pressure on mortality.

**METHODS:**

Mendelian randomization analyses used 125 895 Mexico City Prospective Study participants aged 35 to 74 years at recruitment with valid genetic and other data. Cox regression, adjusted for confounders and regression dilution bias, related blood pressure to mortality in 133 027 participants aged 35 to 74 years without prior chronic disease (other than diabetes) at recruitment.

**RESULTS:**

In the genetic analyses (40 560 [32%] men; mean age 50 years, mean body mass index 29 kg/m^2^) there were 13 153 deaths before age 75 years (3478 cardiovascular, 2053 kidney, and 7622 other). Each 10 mm Hg higher genetically predicted lifelong systolic blood pressure was associated with 73% higher cardiovascular mortality at ages 35 to 74 years (rate ratio, 1.73 [95% CI, 1.44–2.06]), 42% higher kidney death (1.42 [95% CI, 1.15–1.75]), but no clear increase in death from other causes. These lifelong rate ratios were higher than those estimated by observational analyses relating blood pressure in middle age to risk. Mendelian randomization analyses of lifelong diastolic blood pressure confirmed strong associations with cardiovascular but not kidney death. Mortality rate ratios were similar for men and women and in those with versus without diabetes, and broadly similar at different ages and at different proportions of Indigenous American ancestry. Sensitivity analyses gave consistent results.

**CONCLUSIONS:**

In this Mexican population, genetically informed lifelong differences in blood pressure were strongly related to death from cardiovascular and kidney disease.

**NOVELTY AND RELEVANCE**

**What Is New?**

Mendelian randomization analyses have generally been conducted in European-ancestry populations. Here, we conduct Mendelian randomization analyses of blood pressure and cause-specific mortality in the Mexico City Prospective Study, a large blood-based prospective study of Mexican adults.

**What Is Relevant?**

In this population with high prevalences of obesity and diabetes, elevated systolic and diastolic blood pressure were strongly associated with cardiovascular disease mortality. Higher systolic blood pressure was also associated with kidney disease mortality in Mendelian randomization analyses.

**Clinical/Pathophysiological Implications?**

The study reinforces the importance of population-wide and individualized approaches to lower blood pressure in Mexico as well as other populations where obesity and diabetes are common.

According to the Global Burden of Disease Risk Factors Collaboration, elevated systolic blood pressure (SBP), defined as SBP >110 to 115 mm Hg, was responsible for 11 million deaths and 8% of disability-adjusted life years lost in 2021.^1^ In an individual participant meta-analysis of data from 61 prospective cohorts, including 1 million people, each 20 mm Hg lower SBP was associated with a >50% reduction in the stroke death rate, and ≈50% reductions in death rates from ischemic heart disease and other vascular causes.^2^ Evidence on the effects of blood pressure on other diseases, however, have been mixed,^3–5^ perhaps due to reverse causality or residual confounding.^3,6^

Mendelian randomization (MR) studies are increasingly used to evaluate the causal effect of risk factors on disease because, under certain assumptions, they can overcome some inherent biases in traditional prospective observational analyses.^7^ Unlike most observational analyses of cohorts recruited in middle age, they also seek to estimate the effect of a risk factor throughout the entire life-course. An MR study of 380 000 European-ancestry participants from the UK Biobank found that SBP was positively associated with 13 of 20 vascular diseases and with chronic kidney disease (CKD).^8^ However, the generalizability of these findings to non-European ancestry populations, particularly those with different health care systems, risk factor prevalences, and competing risk factors, is uncertain.

Using data from the Mexico City Prospective Study (MCPS),^9^ we previously showed that elevated blood pressure in middle age was strongly associated with vascular and kidney-related mortality, with particularly high absolute excess mortality among individuals with diabetes.^10^ By leveraging the genetic data that now exist in the study,^11^ as well as 5 further years of mortality follow-up, the aim of this report is to compare observational with MR estimates of the effects of blood pressure on vascular, kidney, and other causes of death in this population with high levels of adiposity and diabetes.

## Methods

### Data Availability

Data from the MCPS are available to bona fide researchers for open access or collaboration requests. For more details, the study’s Data and Sample Sharing policy is available (in English and Spanish) at https://www.ctsu.ox.ac.uk/research/mcps. Available study data can be examined in detail through the study’s Data Showcase, available at https://datashare.ndph.ox.ac.uk/mexico/. An Expanded Materials and Methods section with full references^9–31^ is provided in the Supplemental Material; a summary is provided below.

### Study Design, Participants, and Data Collection

Between 1998 and 2004, 159 755 adults aged ≥35 years from the Coyoacán and Iztapalapa districts of Mexico City were recruited into the MCPS.^9^ Baseline data collection included demographic, lifestyle, and participant-reported medical history and medication use, as well as physical measurements and a 10 mL blood sample. Ethics approval was granted by the National Council of Science and Technology in Mexico, the Mexican Ministry of Health, the Ethics and Research commission from the Faculty of Medicine at the National Autonomous University of Mexico, and the University of Oxford.

### Genetic Instruments for Blood Pressure

Participants were genotyped using the Global Screening Array v2 chip from Illumina, as described previously.^11^ Genetic risk scores (GRS) for systolic (SBP-GRS) and diastolic blood pressure (DBP-GRS) were constructed using 1953 of 2103 independent variants identified by the recently reported International Consortium of Blood Pressure and present in the MCPS data set.^15^ Alleles were aligned to be trait-increasing, and GRSs were constructed by multiplying allele counts with relevant weights from the Million Veteran Program trans-ancestry GWAS (genome-wide association study) meta-analysis of SBP or DBP (Supplemental Data Item S1).

### Follow-Up for Mortality

Participants were followed for cause-specific mortality through probabilistic linkage to the Mexican System for Epidemiological Death Statistics (Subsistema Epidemiológico y Estadístico de Defunciones), an electronic death registry in Mexico City administered by the Ministry of Health.^17^ Participant deaths were tracked until September 2022. Table S1 shows the categories of deaths considered in the current report.

### Statistical Methods

All analyses excluded participants aged ≥75 years at recruitment, those with missing or implausible data on blood pressure or other covariates, or those with uncertain mortality linkage. The observational analyses also excluded participants with chronic medical conditions (other than diabetes) to reduce the risk of reverse causality, while the MR analyses excluded those without genetic data passing quality control.^11^

The observational analyses used Cox proportional hazards regression to relate baseline SBP and DBP to cause-specific mortality in models that were stratified by age-at-risk (in 5-year groups) and adjusted for major confounders. These baseline associations were subsequently corrected for regression dilution bias,^19^ as described previously.^10^ The MR analyses are reported in accordance with the Strengthening the Reporting of Observational Studies in Epidemiology using MR guidelines.^20^ An additive genetic model of inheritance was assumed throughout.^21^ To account for the blood pressure-lowering effects of antihypertensive medications,^32^ participants using these medications had their measured SBP increased by 6 mm Hg and DBP increased by 3 mm Hg.^22^ The primary MR analyses used the 1-sample Wald ratio method.^25^ To assess the first MR assumption (relevance), the proportion of variance in baseline SBP and DBP explained by the SBP-GRS and DBP-GRS, as well as *F* statistics,^24^ were estimated. The strength of the association between each GRS and measured blood pressure was also estimated across fifths of the population distribution (ie, across 5 equally sized groups defined by the 4 quintiles of the distribution). The second assumption (independence) was addressed by adjustment for genetic principal components, while the third (exclusion restriction) was assessed first by examining associations with potential confounders and second by performing additional 2-sample MR approaches (including weighted-median,^28^ MR Egger,^29^ and MR Pleiotropy Residual Sum and Outlier^30^). In both the observational and the MR analyses, participants who did not die from the cause of interest were censored in the Cox model at the earliest of death from any other cause, the end of the age-at-risk period of interest, or October 1, 2022. The main analyses are of deaths at ages 35 to 74 years (and deaths before age 75 years are referred to as premature deaths).

Sensitivity analyses included: subdivision of analyses by age, sex, district of residence, previously diagnosed diabetes, and thirds of Indigenous American ancestry^11^ (with tests of heterogeneity across subgroups); use of Steiger filtering^27^ (to minimize reverse causality in analyses of kidney outcomes); restriction to participants unrelated to the third family degree; use of GRSs constructed from studies of populations with admixed American ancestry (rather than trans-ancestry)^16^; and use of a 15/10 mm Hg (rather than a 6/3 mm Hg) adjustment for those on antihypertensive medications at recruitment.

Analyses were conducted with SAS (version 9.4) and R (version 3.3.0).

## Results

### Selection of Study Participants

Of 159 755 recruited participants, 20 689 (13%) were excluded from all analyses. These comprised 7557 (5%) with missing or extreme data, a further 2514 (2%) with uncertain mortality linkage, a further 10 433 (7%) aged ≥75 years at recruitment, and a further 185 (0.1%) who were recruited more than once (data from the first visit at which a blood sample was collected were used for these participants). Of the remaining 139 066 participants, 13 171 (9%) were excluded from MR analyses because they did not have genetic data passing QC criteria, and 6039 (4%) were excluded from observational analyses because they reported diagnoses of chronic diseases (other than diabetes), leaving 125 895 participants in the MR analyses and 133 027 in the observational analyses (Figure S1).

### Validity of the GRS as Instruments for SBP and DBP

Among the 125 895 participants in the MR analyses, the per-allele effects in MCPS of the variants included in the SBP-GRS and DBP-GRS were generally consistent with the estimates provided by the Million Veteran Program trans-ancestry meta-analysis, particularly for SBP (Figure S2). After adjustment for age, age-squared, sex, body mass index, district of residence and genetic PCs, the SBP-GRS and DBP-GRS had *F* statistics of 3715 and 1565, and explained an additional 2.3% and 1.1% of the variance in SBP and DBP, respectively. The Table shows baseline characteristics by fifths of the SBP-GRS distribution. The top fifth of the SBP-GRS distribution had 7.0 mm Hg higher mean SBP and 3.5 mm Hg higher mean DBP than the bottom fifth, while the prevalences of diagnosed hypertension and use of antihypertensive medications between these groups approximately doubled (13.7% versus 26.1% and 9.9% versus 19.9%, respectively). By contrast, the top fifth of the DBP-GRS distribution had 4.0 mm Hg higher mean SBP and 3.1 mm Hg higher mean DBP than the bottom fifth (Table S2).

**Table.**
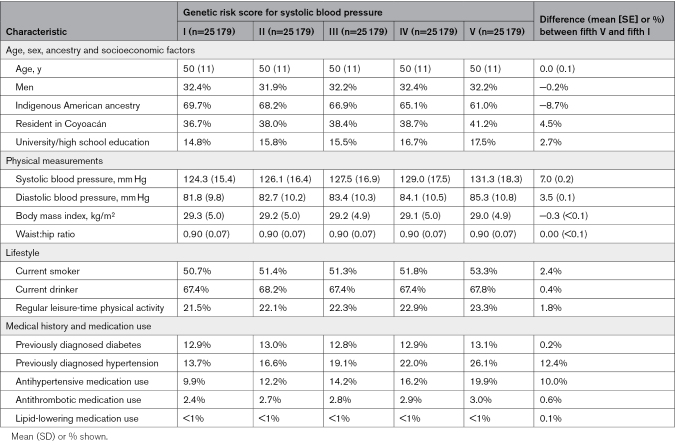
Baseline Characteristics of 125 895 Participants (Genetic Analysis Population) Aged 35 to 74 Years at Recruitment by Fifth of Genetic Risk Score for Systolic Blood Pressure

**Figure 1.**
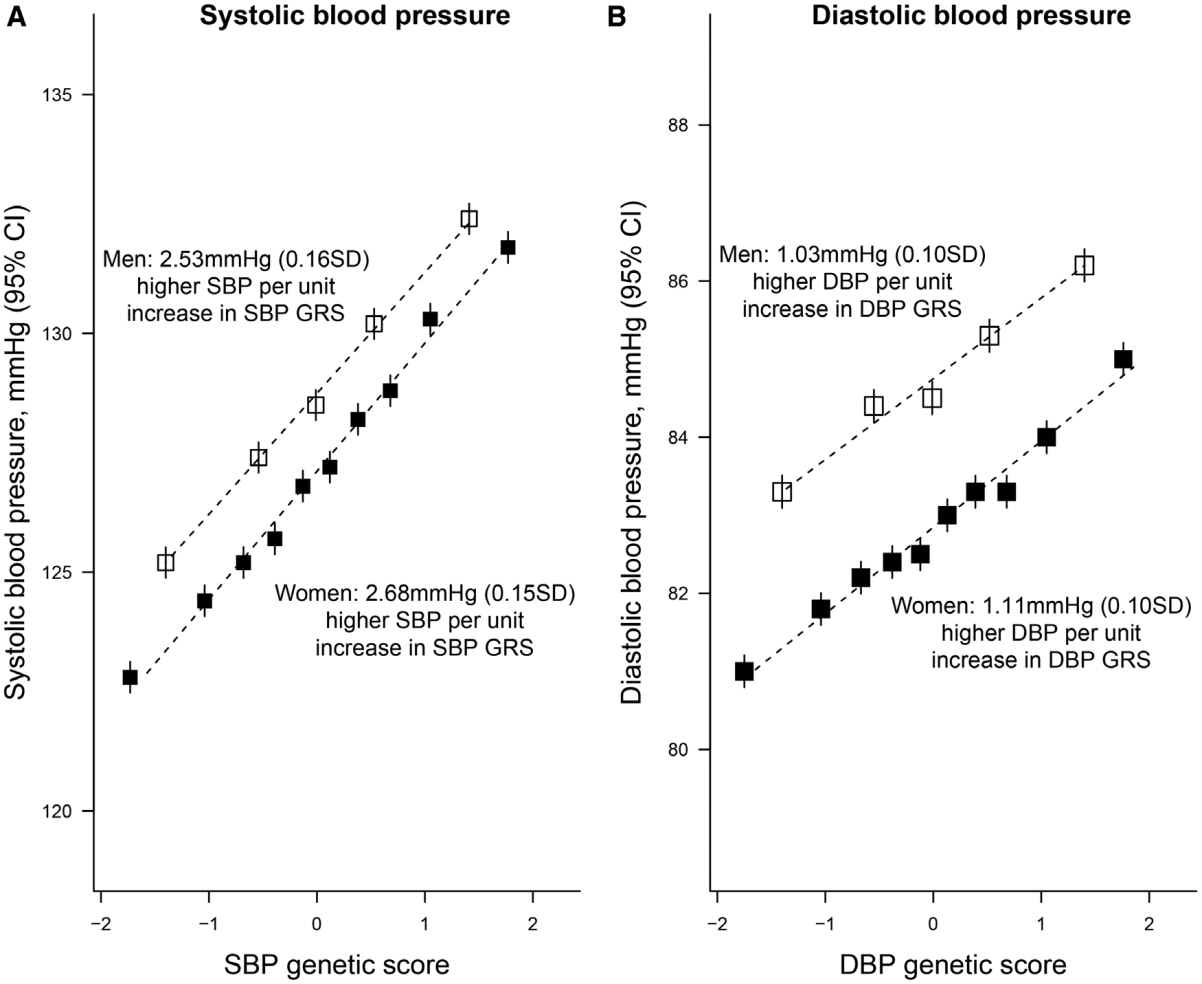
**Adjusted mean systolic blood pressure (SBP) and diastolic blood pressure (DBP) by genetic score groups in men and women.** In each panel, 10 equally sized groups are shown for women and 5 for men so that each point includes ≈8000 participants. Each unit represents 1 SD. All estimates are adjusted for age, age-squared, body mass index, district of residence and 7 genetic principal components. GRS indicates genetic risk score; and RR, rate ratio.

Each 1 SD higher SBP-GRS was associated with 2.6 mm Hg higher SBP (equivalent to 0.15 SD higher SBP) while each 1 SD higher DBP-GRS was associated with 1.1 mm Hg higher DBP (0.10 SD); these estimates were nearly identical in men and women (Figure 1) and when estimated separately by age, district of residence, or level of Indigenous American ancestry (Figures S3 and S4). Most other risk factors were similar across the SBP-GRS groups, although participants in the lowest fifth of SBP-GRS had a higher average proportion of Indigenous American ancestry and were less likely to live in Coyoacán (the wealthier of the 2 districts). Baseline characteristics of the 133 027 participants included in the observational analyses are shown in Tables S3 and S4.

### Associations of SBP and DBP With Cardiovascular and Kidney Disease Mortality

**Figure 2.**
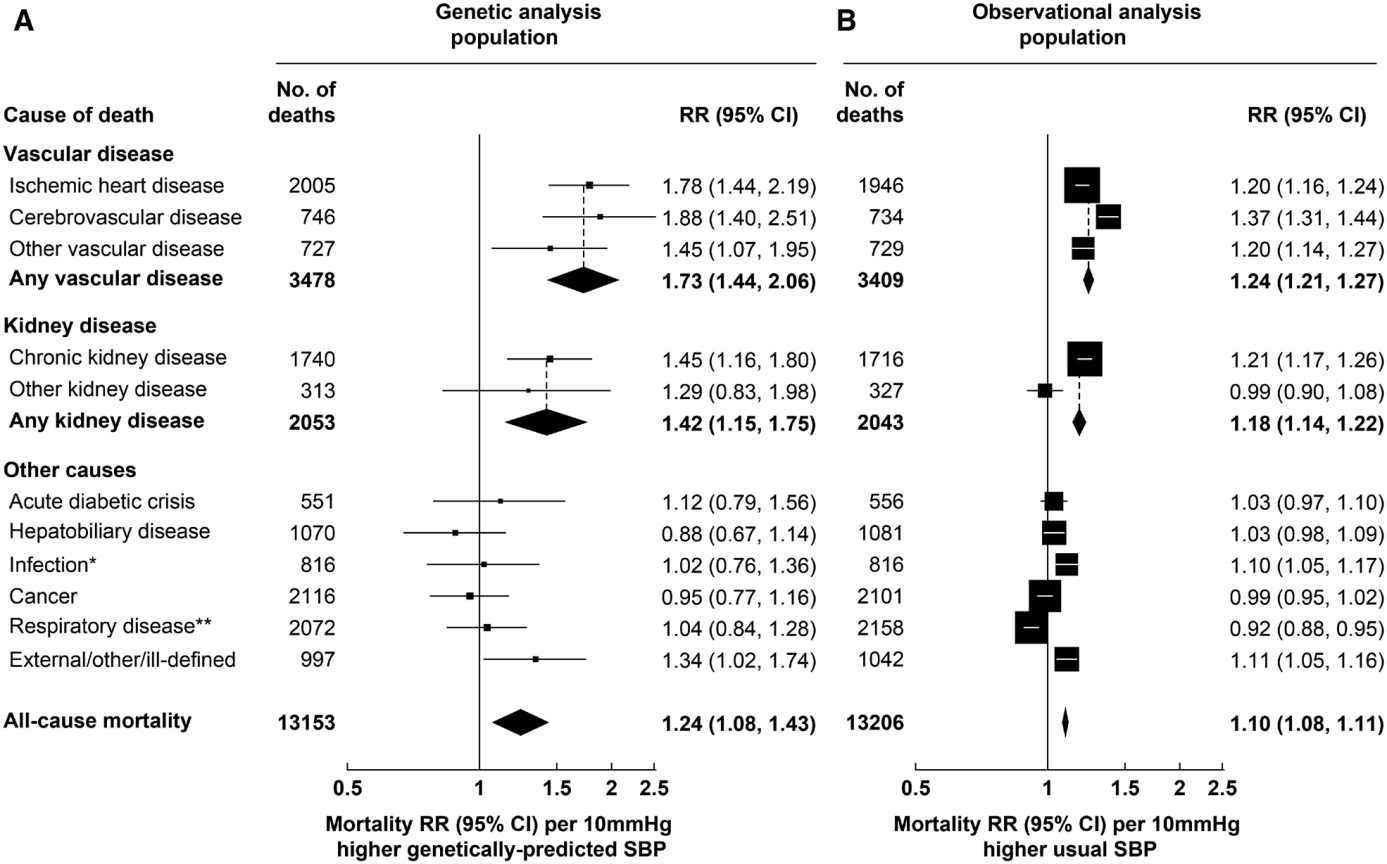
**Genetic and observational associations of systolic blood pressure (SBP) with cause-specific mortality.** Genetic analyses are stratified by age-at-risk and adjusted for biological sex, body mass index, district of residence, and 7 genetic principal components. Observational analyses are stratified by age-at-risk and adjusted for sex, district, educational level, smoking status, diabetes status, alcohol drinking, leisure-time physical activity, height, weight, waist circumference and hip circumference, and are adjusted for regression dilution bias. The area of each box is inversely proportional to the variance of the log rate ratio (RR). *Excludes respiratory infections. **Includes respiratory infections.

**Figure 3.**
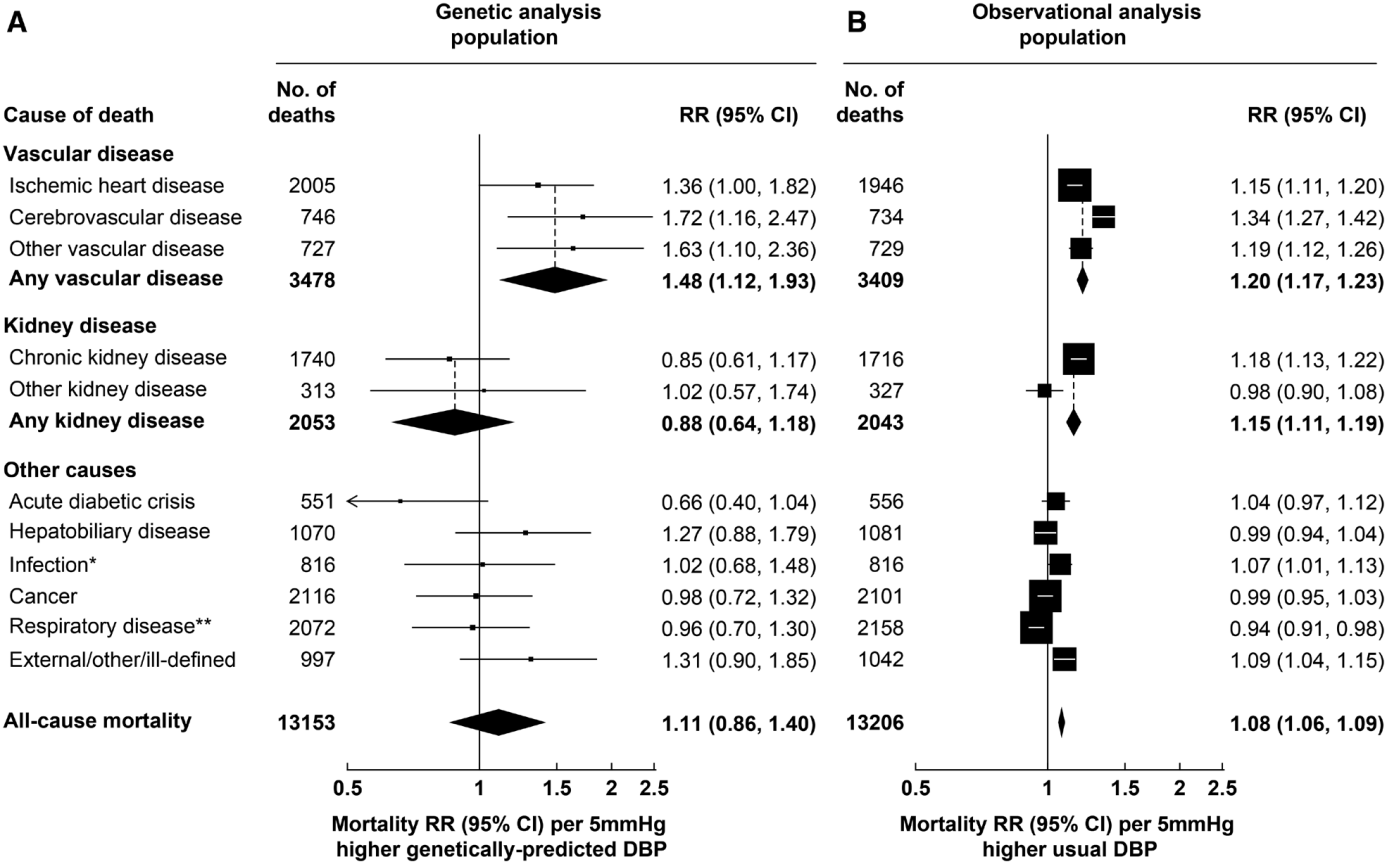
**Genetic and observational associations of diastolic blood pressure (DBP) with cause-specific mortality.** Genetic analyses are stratified by age-at-risk and adjusted for biological sex, body mass index, district of residence, and 7 genetic principal components. Observational analyses are stratified by age-at-risk and adjusted for sex, district, educational level, smoking status, diabetes status, alcohol drinking, leisure-time physical activity, height, weight, waist circumference and hip circumference, and are adjusted for regression dilution bias. The area of each box is inversely proportional to the variance of the log rate ratio (RR). *Excludes respiratory infections. **Includes respiratory infections.

During a median follow-up in survivors of 20 years, there were 3478 vascular deaths at ages 35 to 74 years in the MR analysis population and 3409 vascular deaths in the observational analysis population (Figure 2). Each 10 mm Hg higher genetically predicted SBP was associated with 73% higher vascular mortality risk (rate ratio [RR], 1.73 [95% CI, 1.44–2.06]). The RR estimates were similar when separately considering ischemic heart disease (RR, 1.78 [95% CI, 1.44–2.19]), cerebrovascular disease (RR 1.88, 1.40–2.51) and other vascular disease (RR 1.45, 1.07–1.95). These associations were substantially stronger than those arising from the observational analyses relating usual SBP in middle age to mortality risk, where each 10 mm Hg higher usual SBP was associated with 24% higher overall vascular mortality risk (RR 1.24, 1.21–1.27). Each 5 mm Hg higher genetically predicted DBP was associated with 48% higher vascular mortality risk (RR 1.48, 1.12–1.93; Figure 3). As for SBP, this association was similar for specific vascular causes and substantially stronger than those arising from the observational analyses relating usual DBP in middle age to risk (where the vascular mortality RR was 1.20 [95% CI, 1.17–1.23]).

There were 2053 kidney disease deaths at ages 35 to 74 years in the MR analysis population (2043 in the observational analysis population), most of which were due to CKD. Each 10 mm Hg higher genetically predicted SBP was associated with 42% higher kidney disease mortality risk (RR, 1.42 [95% CI, 1.15–1.75]; Figure 2). This association was also substantially stronger than the association arising from the observational analyses relating usual SBP to kidney disease mortality (RR, 1.18 [95% CI, 1.14–1.22]). By contrast, although each 5 mm Hg higher DBP was associated with an increased risk of kidney disease mortality in the observational analyses (RR, 1.15 [95% CI, 1.11–1.19]) it was not in the MR analyses (RR, 0.88 [95% CI, 0.64–1.18; Figure 3).

**Figure 4.**
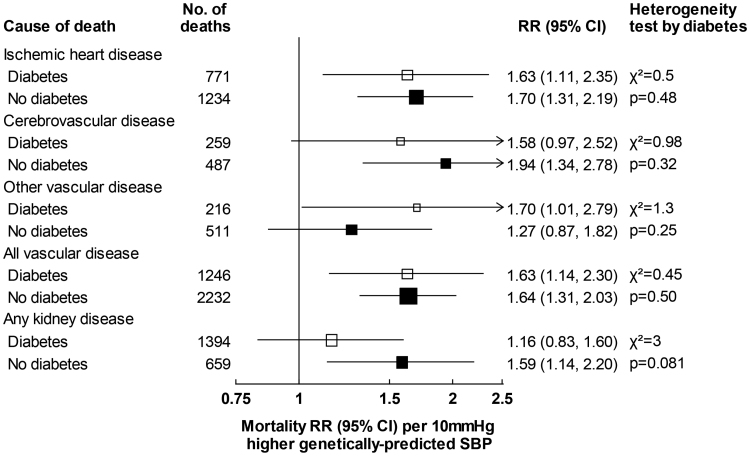
**Associations between genetically predicted systolic blood pressure (SBP) and vascular and kidney mortality by diabetes status.** Rate ratios (RRs) per 10 mm Hg higher genetically predicted SBP are stratified by age-at-risk and adjusted for biological sex, body mass index, district of residence, and 7 genetic principal components. Heterogeneity is assessed using Cochran *Q* statistic.

For both SBP and DBP, associations of genetically predicted values with vascular and kidney disease mortality were similar among those with and without diabetes (Figure 4; Figure S5). For example, each 10 mm Hg higher genetically predicted SBP was associated with 63% higher vascular mortality risk in those with diabetes (RR, 1.63 [95% CI, 1.14–2.30]) and 64% higher vascular mortality risk in those without diabetes (RR, 1.64 [95% CI, 1.31–2.03]).

### Associations With Other Causes of Death

In the MR analysis population, there were 7622 nonvascular nonkidney disease deaths, including 2116 cancer deaths, 2072 respiratory deaths, 1070 deaths from hepatobiliary disease, 816 infection deaths, 551 deaths from an acute diabetic crisis, and 997 deaths from other causes. There was no strong evidence that genetically predicted SBP or DBP was associated with any of these categories of death (Figures 2 and 3).

### Sensitivity Analyses

Removal from the GRSs of 76 variants that had a statistically significantly stronger effect on estimated glomerular filtration rate (eGFR) than on blood pressure (Steiger filtering) had little effect on the magnitude of associations seen between genetically predicted SBP and DBP with specific causes of death (Figures S6 and S7). Similarly, for vascular and kidney mortality, analyses stratified by age (Figures S8 and S9), biological sex (Figures S10 and S11), district of residence (Figures S12 and S13), or thirds of Indigenous American ancestry (Figures S14 and S15) showed broadly consistent effects in all subgroups. Analyses using GRSs constructed with admixed American weights rather than the trans-ancestry weights (Figures S16 and S17), or that were performed in the subset of participants unrelated to the third degree (Figures S18 and S19), or that used a 15/10 mm Hg SBP/DBP adjustment for antihypertensive use (Figure S20) also did not differ much from the main analyses. Results using 2-sample MR approaches were directionally similar to those from the main analyses (Tables S5 and S6), but the magnitudes of associations were slightly smaller. There was no clear evidence of horizontal pleiotropy with the weighted-median estimates being consistent with the inverse variance-weighted estimates and the MR Egger intercepts not being significantly different from zero. MR Pleiotropy Residual Sum and Outlier identified a single outlier for analyses of premature death due to CKD, and 2 for analyses of all-cause mortality. Their removal had no material effect on the results.

## Discussion

In this large, admixed Mexican population with high levels of obesity and diabetes we found strong evidence supporting a causal relationship between blood pressure and death from vascular and kidney disease. Each 10 mm Hg higher genetically predicted lifelong SBP was associated with a 73% increase in the risk of vascular death and 42% increase in the risk of kidney death (chiefly from CKD), while each 5 mm Hg higher genetically predicted lifelong DBP was associated with a 48% increase in the risk of vascular death but no apparent increase in risk of kidney death. These associations were similar in those with and without diabetes, meaning that the absolute lifelong relevance of higher blood pressure to risk was much greater in those with diabetes. The genetic associations were also similar irrespective of age, sex, or proportion of Indigenous American ancestry.

By contrast with observational studies that tend to examine the relationship between a risk factor measured in middle age and the subsequent risk of disease, MR analyses aim to estimate the lifelong causal effect of a risk factor.^33^ This may explain why we observed larger relative risk estimates in the MR analyses compared with those from the observational analyses. One previous study found that genetically predicted SBP was associated not only with SBP but also with the rate of increase in SBP with age.^34^ This may also help explain why MR estimates of the effects of blood pressure on risk tend to be larger than those from observational studies. In a previous observational analysis from the Prospective Studies Collaboration of individual-participant-data from 61 prospective studies, each 10 mm Hg higher SBP was associated with 35% higher risk of ischemic heart disease mortality and 50% higher risk of stroke mortality at ages 40 to 79 years^2^ (both slightly higher than our observational estimates). By contrast, a MR study of 380 000 participants of European ancestry from the UK Biobank found that each 10 mm Hg increase in SBP was associated with a 59% increase in the odds of ischemic heart disease and 52% increase in the odds of ischemic cerebrovascular disease (both slightly lower than our MR relative risk estimates).^8^ The UK Biobank MR study also found 10 mm Hg higher SBP to be associated with a 39% increase in the odds of CKD (very similar to the 42% increase in our report). Other MR analyses in UK Biobank have confirmed the genetic association of SBP with both reduced eGFR and albuminuria.^26,35^ However, to date, there have been too few cases of end stage kidney disease in UK Biobank to reliably estimate its genetic associations. Our approach to correcting for regression dilution bias based on an estimate taken at the midpoint of the period between the baseline and the resurvey assessments was likely to be somewhat conservative, as previous studies have found that most of the long-term regression to the mean in blood pressure occurs shortly after the baseline measurement.^19^ Other theoretical explanations for the larger RRs seen in our MR analyses compared with our observational analyses include pleiotropic effects of the genetically predicted SBP and DBP, or potential inflation due to the high level of relatedness within the MCPS cohort. However, our 2-sample MR analyses provided no evidence for pleiotropy, while our results were similar when repeated in the subset of participants who were unrelated to the third degree.

Randomized trials corroborate the findings that associations between blood pressure and cardiovascular diseases are causal by demonstrating that lowering blood pressure lowers risk. Such trials have confirmed that reducing SBP leads to reductions in the subsequent risk of major cardiovascular events, including ischemic heart disease, stroke and heart failure. For example, in a meta-analysis of 123 randomized trials of blood pressure-lowering treatments, each 10 mm Hg reduction in SBP reduced the risk of coronary heart disease by 17% (95% CI, 12%–22%), the risk of stroke by 27% (13%–32%), and the risk of heart failure by 28% (12%–33%),^5^ which is in line with the estimates from the observational analyses in this report.

For kidney failure, these trials found a nonsignificant 5% reduction in risk for each 10 mm Hg reduction in SBP. However, median follow-up in those trials was only 3 years and CKD has a long latent phase. A meta-analysis of 2 blood pressure-lowering trials including 1907 participants with CKD followed for a median of 15 years suggested a benefit; strict blood pressure control led to a borderline reduction in kidney failure risk of 12% (95% CI, 0%–22%).^4^ The differing results from our MR analyses of SBP and DBP on kidney disease mortality support previous hypotheses that glomerular barotrauma is a key mechanism through which blood pressure affects kidney disease risk. Specifically, the findings are consistent with the view that elevated SBP may increase the pressure difference across the glomerulus, whereas higher DBP may reduce it, thereby lowering glomerular sheer stress.^26^ Reducing the risk of kidney failure is especially important in settings where access to treatments such as dialysis and transplantation are very limited, such as Mexico.^36^

The findings in the current report have public health and policy implications not just for Mexico but also for other populations, including the millions of Mexican-Americans living in the United States, where obesity and diabetes are common and the absolute risks of cardiovascular and kidney disease are high. Mexican^37^ and US^38^ guidelines advise targeting a blood pressure <140/90 mm Hg, or <130/80 mm Hg for those with previous cardiovascular disease or a >10% predicted 10-year risk of cardiovascular disease. Our findings suggest that successful implementation of these guidelines, together with population-wide approaches that deliver reductions in mean blood pressure throughout the whole population, could greatly reduce the burden of preventable disease on both health care systems and individuals in Mexico and elsewhere.

Together with its large size and prolonged duration of follow-up, a key strength of the present study is that it extends the validity of previous findings beyond high-income populations of predominantly European ancestry to a Latin American population with high prevalences of obesity and diabetes. The availability of genetic data in all participants allowed the use of a 1-sample MR approach where instrument-to-BP and instrument-to-mortality associations are estimated in the same underlying population. The use of a single allele score in preference to each genetic variant acting as a separate instrumental variable (as done in one of the sensitivity analyses) also helps reduce any weak instrument bias.^39^ The primary instrument was derived from a trans-ancestry GWAS meta-analysis and there was no overlap between MCPS participants and participants in the studies used to identify the variants in the GRS or determine their weights. The strength of associations between the instruments and blood pressure in the current study were clear, consistent and similar among different types of individuals.

Limitations of the present study include the potential for some horizontal pleiotropy, although sensitivity analyses involving a range of alternative MR approaches yielded consistent results. We have not explored mediation pathways, while the recruitment of participants aged ≥35 years means that we cannot explore the role of blood pressure on diseases at younger ages. The performance of the GRS would likely be improved with additional admixed American ancestry data from blood pressure GWAS. The study population arises from 2 districts of Mexico City, and so the participants are not representative of all adults throughout Mexico^40,41^ (or even Mexico City) with respect to socioeconomic risk factors, including access to health care, or with respect to lifestyle risk factors. However, prospective studies of nonrepresentative cohorts of individuals can provide reliable evidence about the associations of risk factors with disease that are widely generalizable^42,43^ and, when interpreted in conjunction with national age- and sex-specific mortality rates, can be used to estimate the absolute excess risks of specific causes of death beyond the immediate study population. Finally, a lack of information on nonfatal outcomes means that the conclusions apply directly only to causes of death.

## Conclusions

In this large study of a Mexican population with high levels of obesity and diabetes, blood pressure was strongly associated with cardiovascular and kidney mortality. The findings reinforce the need in Mexico (and in other populations where obesity and its downstream effects are common) for the combination of both population-wide and individualized approaches to reduce blood pressure and the risk of cardiovascular and kidney disease.

## Perspectives

In this prospective study of adults from Mexico City, both SBP and DBP displayed strong positive associations with cardiovascular mortality in both the MR and the conventional observational analyses. MR analyses also suggested that SBP, but not DBP, was causally related to kidney mortality, perhaps due to a mediating effect of glomerular barotrauma. The relative effects of blood pressure on mortality were similar in those with and without diabetes, meaning that the absolute effects of higher blood pressure on risk were greater for those with diabetes. The findings reinforce the need for effective strategies to improve blood pressure control in Mexico, as well as in other populations where obesity and diabetes are common. Further MR analyses in diverse populations are needed to establish the full effects of blood pressure on morbidity and mortality across different socioeconomic and health care settings.

## ARTICLE INFORMATION

### Acknowledgments

The authors thank the study participants and staff.

### Author Contributions

Establishing the cohort: P. Kuri-Morales, J. Alegre-Díaz, and R. Tapia-Conyer. Obtaining funding and enhancing the cohort: P. Kuri-Morales, R. Collins, R. Peto, J.R. Emberson, J. Alegre-Díaz, and R. Tapia-Conyer. Data acquisition, analysis, or interpretation of data: All authors. Drafting first version of article: M. Turner. Critical revision of the report for important intellectual content: All authors. All authors have seen and approved the final version and agreed to its publication. J.R. Emberson and J. Alegre-Díaz had full access to all the data in the study, verified the underlying data, take responsibility for the integrity of the data and the accuracy of the analysis, and took the decision to submit.

### Sources of Funding

The Mexico City Prospective Study has received funding from the Mexican Health Ministry, the National Council of Science and Technology for Mexico, Wellcome (058299/Z/99), Cancer Research UK, British Heart Foundation (RE/13/1/30181), Kidney Research UK (MR/R007764/1), and the UK Medical Research Council (MC_UU_00017/2, MR/Z504543/1). The funding sources had no role in the design, conduct or analysis of the study or the decision to submit the article for publication.

### Disclosures

F. Bragg reports funding from a UK Medical Research Council grant to Health Data Research UK (HDR-23007; Molecules to Health Records). R. Collins reports grants to the University of Oxford from AstraZeneca and Regeneron Pharmaceuticals, having a patent for a statin-related myopathy genetic test licensed to the University of Oxford from Boston Heart Diagnostics (R. Collins has waived any personal reward with any share in royalty and other payments waived in favor of the Nuffield Department of Population Health, University of Oxford) and being deputy chair of not-for-profit clinical trial company PROTAS, Chief Executive of UK Biobank, and Chair of the steering committee of the ORION-4 clinical trial of inclisiran. N. Staplin, W.G. Herrington, and R. Haynes report grants to the University of Oxford from Boehringer Ingelheim and Eli Lilly. N. Staplin additionally reports grants to the University of Oxford from Novo Nordisk. W.G. Herrington was supported by a Medical Research Council Kidney Research UK Professor David Kerr Clinician Scientist Award (MR/R007764/1). J.R. Emberson reports grants to the University of Oxford from AstraZeneca, Regeneron Pharmaceuticals, Boehringer Ingelheim, and Eli Lilly. The other authors report no conflicts.

### Supplemental Material

Expanded Materials and Methods

Tables S1–S6

Figures S1–S20

Supplemental Data Item S1

## Supplementary Material


