# Supplementary material for: Blood Pressure and Mortality in Mexico City: A Mendelian Randomization Study
Source: Hypertension. 2025 Sep 17;82(11):1896–905. doi: 10.1161/HYPERTENSIONAHA.125.25348 (PMC7618184; doi:10.1161/HYPERTENSIONAHA.125.25348)
Supplement: Supplementary file 1 [file hyp-82-1896-s001.pdf]

Supplemental Material

Blood pressure and cause-specific mortality in Mexico City: a Mendelian randomization study

Michael Turner[1]*, Pablo Kuri-Morales[2,3]*, Jesús Alegre-Díaz[4]*, Paulina Baca[4], Jose Adrián Garcilazo-Ávila[4], Carlos González-Carballo[4], Raul Ramirez-Reyes[4], Fernando Rivas[4], Diego Aguilar-Ramírez[1], Fiona Bragg[1,5], Louisa Gnatiuc Friedrichs[1], William G Herrington[1], Michael Hill[1], Eirini Trichia[1], Alejandra Vergara-Lope[1], Rachel Wade[1], Doreen Zhu[1], Rory Collins[1], Richard Peto[1], Jaime Berumen[4], Natalie Staplin[1], Jason Torres[1], Richard Haynes[‡1], Jonathan R Emberson[†‡1], Roberto Tapia-Conyer[‡3]

* Joint first authors. † Corresponding author. ‡ Joint last authors.

[1]Clinical Trial Service Unit and Epidemiological Studies Unit, Nuffield Department of Population Health (NDPH), University of Oxford, UK; [2]Instituto Tecnológico y de Estudios Superiores de Monterrey, Monterrey, Mexico; [3]Faculty of Medicine, UNAM, Mexico City, Mexico; [4]Experimental Research Unit, Faculty of Medicine, National Autonomous University of Mexico (UNAM), Mexico City; [5]Health Data Research UK Oxford, University of Oxford, Oxford, UK.

Correspondence:
Professor Jonathan Emberson
Clinical Trial Service Unit and Epidemiological Studies Unit, Big Data Institute, Old Road Campus, Roosevelt Drive, Oxford, OX3 7LF, United Kingdom
email: jonathan.emberson@ndph.ox.ac.uk

Supplemental Material Table of Contents

Page

Expanded materials and methods 3

References 6

Supplemental tables

Genetic analysis population (n=125 895 participants aged 35-74 years)

S1 Number of deaths at ages 35-74 years by underlying cause 8
S2 Baseline characteristics by fifth of genetic risk score for DBP 10

Observational analysis population (n=133 212 participants aged 35-74 years)

S3 Baseline characteristics by baseline systolic blood pressure 11
S4 Baseline characteristics by baseline diastolic blood pressure 12

Genetic associations with cause-specific mortality using robust Mendelian randomisation methods based on summary-level data

S5 Systolic blood pressure 13
S6 Diastolic blood pressure 14

Supplemental figures

S1 Participant flow diagram 15
S2 MCPS vs MVP variant weights for: a) SBP; and b) DBP 16
S3 Adjusted mean SBP by genetic score by age, district and ancestry 17
S4 Adjusted mean DBP by genetic score by age, district and ancestry 18
S5 Genetically-predicted DBP and cardiovascular and kidney mortality by diabetes status 19

Sensitivity analyses

S6 Steiger filtering, SBP 20
S7 Steiger filtering, DBP 21
S8 Analyses by age, SBP 22
S9 Analyses by age, DBP 23
S10 Analyses by sex, SBP 24
S11 Analyses by sex, DBP 25
S12 Analyses by district, SBP 26
S13 Analyses by district, DBP 27
S14 Analyses by ancestry, SBP 28
S15 Analyses by ancestry, DBP 29
S16 Use of an IAM-specific vs trans-ancestry instrument, SBP 30
S17 Use of an IAM-specific vs trans-ancestry instrument, DBP 31
S18 Restriction to unrelated subset, SBP 32
S19 Restriction to unrelated subset, DBP 33
S20 Use of 15/10 mmHg correction for antihypertensive use (rather than 6/3 mmHg) 34

Expanded Materials and Methods

Study design, participants and data collection

Between 1998 and 2004, 159 755 adults aged ≥35 years from the Coyoacán and Iztapalapa districts of Mexico City were recruited into the Mexico City Prospective Study.[1] Baseline data collection included demographic, lifestyle, and participant-reported medical history and medication use. Seated SBP and diastolic blood pressure (DBP) were measured 3 times (at 1-minute intervals) to the nearest 1mmHg using a standard mercury sphygmomanometer. Weight, height, waist and hip circumference were measured, and a 10 mL non-fasting venous blood sample was collected. Body mass index (BMI) was calculated as the weight in kg divided by the square of height in meters. Hemoglobin A1c (HbA_{1c}) was measured from the buffy coat sample using validated high-performance liquid chromatography methods[2] on HA-8180 analyzers (Arkray Inc) with calibrators traceable to International Federation of Clinical Chemistry and Laboratory Medicine standards.[3] Between 2015 and 2019, 10 144 surviving participants were revisited in their homes and agreed to take part in a resurvey, which involved similar procedures as the baseline assessment. Ethics approval was granted by the National Council of Science and Technology in Mexico, the Mexican Ministry of Health, the Ethics and Research commission from the Medicine Faculty at the National Autonomous University of Mexico, and the University of Oxford.

Genetic instruments for blood pressure

Participants were genotyped using the Global Screening Array v2 chip from Illumina, as described previously.[4] Imputation was conducted using the Trans-Omics Precision Medicine (TOPMed) version R2 in the GRCh38 imputation server[5] and variants with imputation r^2 >0.4 were considered valid for these analyses. Genetic risk scores for SBP (SBP-GRS) and DBP (DBP-GRS) were constructed using the 2103 independent (LD r^2<0.1, per Keaton *et al.*[6]) variants association with blood pressure traits recently reported by the International Consortium of Blood Pressure genome-wide association study (GWAS).[6] These variants were identified primarily in studies restricted to European-ancestry participants, but their weights were taken from the trans-ancestry meta-analysis GWAS results from the Million Veteran Program (MVP)[7]. Of these 2103 variants, 52 were not present in either MVP or MCPS and 98 were ambiguous (defined as an A/T or C/G variant with minor allele frequency ≥0.42), leaving 1953 for SBP-GRS and DBP-GRS construction. Alleles were aligned to be trait-increasing in MVP and each weighted GRS was constructed by multiplying individual allele counts with the relevant weight (**Supplemental Data Item 1**).

Follow-up for mortality

Death registration in Mexico City is reliable and complete, with almost all deaths medically certified.[8] Participants are followed for cause-specific mortality through probabilistic linkage based on name (including phonetic coding of names), age and sex to the Mexican System for Epidemiologic Death Statistics (*Subsistema Epidemiológico y Estadístico de Defunciones* [SEED]), an electronic death registry in Mexico City administered by the Ministry of Health. Field validation of more than 7000 deaths matched in this way confirmed the reliability of the algorithm in more than 95% of cases. Diseases recorded on death certificates were coded using the International Statistical Classification of Diseases and Related Health Problems, Tenth Revision (ICD-10), with subsequent review by study clinicians to recode, where necessary, the underlying cause of death.[9] Participant deaths were tracked until 1st October 2022. **Table S1** shows the categories of deaths considered in the current report.

Statistical methods

All analyses excluded participants aged ≥75 years at recruitment, those with missing or implausible data on blood pressure (SBP >250 or <80mmHg, DBP >150 or <40mmHg, or pulse pressure <15mmHg) or any covariate (see below), or with uncertain mortality linkage (defined as a ≥1 year discrepancy in a participant's date of birth as recorded at the baseline

survey compared with the matched death certificate). For the observational analyses, those with any chronic medical condition (other than diabetes) were also excluded to reduce the risk of reverse causality bias, which can happen when pre-existing diseases change baseline blood pressure and thereby distort the true prospective relationship between blood pressure and mortality risk. For the MR analyses, those without genotype array data passing QC[4] were excluded.

The associations between measured SBP and DBP at recruitment and cause-specific mortality were assessed in Cox proportional hazard models stratified by age-at-risk (in five year groups), and adjusted for sex, anthropometry (height, weight, and waist and hip circumference, each split into four equally sized groups), education (university/college/high school, middle school, elementary school, or other), district of residence (two districts), smoking status (never, former, occasional, <10 cigarettes per day, ≥10 cigarettes per day), alcohol consumption (none, former use, current use), leisure-time physical activity (none, up to twice weekly, at least three times weekly), and diabetes status (divided into five groups: no diabetes; undiagnosed diabetes [ie, no previous diagnosis but HbA_{1c} ≥6.5%]; previously diagnosed diabetes with HbA_{1c} <9%; previously diagnosed diabetes with HbA_{1c} ≥9% to <11%; and previously diagnosed diabetes with HbA_{1c} ≥11%). The hazard ratios from these observational analyses relating baseline blood pressure to risk were subsequently corrected for regression dilution bias,[10] by dividing both the log hazard ratio and its standard error by an estimate of the regression dilution ratio at around midpoint of the period between recruitment and the 15-year resurvey.[11] This correction is likely to be somewhat conservative, however, as previous studies have found that most of the long-term regression to the mean in blood pressure occurs shortly after an initial baseline assessment.[10]

The MR analyses are reported in accordance with Strengthening the Reporting of Observational Studies in Epidemiology using Mendelian Randomization (STROBE-MR) guidelines.[12] As done previously,[13] an additive genetic model of inheritance was assumed throughout. The three core MR assumptions are that the genetic score: (1) predicts the exposure of interest (relevance); (2) is not associated with genetic confounding factors (independence); and (3) is only associated with the outcome via the exposure of interest (exclusion restriction). To account for the blood pressure lowering effects of antihypertensive medications, those reportedly on such a medication at recruitment had their measured SBP increased by 6mmHg and their DBP increased by 3mmHg (based on recent estimates of the long-term effect of antihypertensive medications).[14] The proportion of SBP and DBP variance explained by the SBP-GRS and DBP-GRS (r^2) was estimated from the increase in r^2 observed when each instrument was incorporated into a linear model adjusted for age, age-squared, sex, BMI, district of residence and the first seven genetic principal components (PCs) to capture population stratification.[15] The F-statistic was calculated from the square of the t-value from the linear regression model.[16] The primary MR analyses employed the one-sample Wald ratio method,[17] in which the association of the SBP-GRS with SBP (or the DBP-GRS with DBP) was estimated using linear regression after adjustment for age, age-squared, sex, BMI (for consistency with most previous reports[13,18]), district of residence and genetic PCs, and the association between the SBP-GRS (or the DBP-GRS) and cause-specific mortality was estimated using Cox proportional hazards models, stratified by age-at-risk (in five-year groups), and adjusted for sex, BMI, district of residence, and genetic PCs.

In both the observational and the MR analyses, participants who did not die from the cause of interest were censored at the earliest of death from any other cause, the end of the age-at-risk period of interest, or 1 October 2022. The main analyses are of deaths at ages 35-74 years (which are referred to as 'premature' deaths).

Sensitivity analyses included: analyses of mortality by age, sex, district of residence, previously-diagnosed diabetes and thirds of Indigenous American (IAM) ancestry[4]; use of

Steiger filtering[19] to exclude from the GRS any variants for which (in the MVP GWAS)[7] the proportion of variance explained for estimated glomerular filtration rate (eGFR) was statistically significantly greater than that for blood pressure (to mitigate the influence of effects of variants on blood pressure being mediated by kidney function); restriction to participants unrelated to the 3rd family degree; use of alternative GRSs constructed with weights solely from MVP individuals of admixed AMR ancestry[7]; and use of a 15/10mmHg adjustment (rather than a 6/3mmHg) adjustment for those on antihypertensive medications at recruitment.

To examine the possibility of horizontal pleiotropy in the MR analyses we performed additional 'two-sample' MR approaches. The weighted-median method[20] is robust to horizontal pleiotropy assuming the majority of variants are valid instruments. Inverse variance-weighted (IVW) MR was also performed because the weighted median method requires a comparator and IVW is the standard two-sample method. MR Egger[21] detects and assesses the significance of horizontal pleiotropy by relaxing the assumption from the IVW method that only the risk factor of interest is mediating the effects on the outcome. MR-PRESSO[22] detects and removes outlier variants. For two-sample methods, associations between individual variants and SBP or DBP were taken from the Million Veteran Program results as for the weights in the primary analysis GRSs. Associations between individual variants and mortality outcomes were assessed using the --coxph option in SUGEN[23] adjusting for age, age-squared, biological sex, BMI, district of residence and genetic PCs.

References

1. Tapia-Conyer R, Kuri-Morales P, Alegre-Díaz J, et al. Cohort Profile: The Mexico City Prospective Study. *Int J Epidemiol*. 2006;35(2):243-249. doi:10.1093/ije/dyl042

2. Youngman LD, Clark S, Manley S, Peto R, Collins R. Reliable Measurement of Glycated Hemoglobin in Frozen Blood Samples: Implications for Epidemiologic Studies. *Clin Chem*. 2002;48(9):1627-1629. doi:10.1093/clinchem/48.9.1627

3. National Glycohemoglobin Standardization Program. International Federation of Clinical Chemistry standardization of HbA1c. Available at: https://ngsp.org/ifccngsp.asp. Accessed September 16, 2025.

4. Ziyatdinov A, Torres J, Alegre-Díaz J, et al. Genotyping, sequencing and analysis of 140,000 adults from Mexico City. *Nature*. 2023;622(7984):784-793. doi:10.1038/s41586-023-06595-3

5. Taliun D, Harris DN, Kessler MD, et al. Sequencing of 53,831 diverse genomes from the NHLBI TOPMed Program. *Nature*. 2021;590(7845):290-299. doi:10.1038/s41586-021-03205-y

6. Keaton JM, Kamali Z, Xie T, et al. Genome-wide analysis in over 1 million individuals of European ancestry yields improved polygenic risk scores for blood pressure traits. *Nat Genet*. 2024;56(5):778-791. doi:10.1038/s41588-024-01714-w

7. Verma A, Huffman JE, Rodriguez A, et al. Diversity and scale: Genetic architecture of 2068 traits in the VA Million Veteran Program. *Science*. 2024;385(6706):eadj1182. doi:10.1126/science.adj1182

8. Mikkelsen L, Phillips DE, AbouZahr C, et al. A global assessment of civil registration and vital statistics systems: monitoring data quality and progress. *The Lancet*. 2015;386(10001):1395-1406. doi:10.1016/S0140-6736(15)60171-4

9. Alegre-Díaz J, Herrington W, López-Cervantes M, et al. Diabetes and Cause-Specific Mortality in Mexico City. *N Engl J Med*. 2016;375(20):1961-1971. doi:10.1056/NEJMoa1605368

10. Clarke R, Shipley M, Lewington S, et al. Underestimation of risk associations due to regression dilution in long- term follow-up of prospective studies. *Am J Epidemiol*. 1999;150(4):341-353. doi:10.1093/oxfordjournals.aje.a010013

11. Tapia-Conyer R, Alegre-Díaz J, Gnatiuc L, et al. Association of Blood Pressure With Cause-Specific Mortality in Mexican Adults. *JAMA Netw Open*. 2020;3(9):e2018141. doi:10.1001/jamanetworkopen.2020.18141

12. Skrivankova VW, Richmond RC, Woolf BAR, et al. Strengthening the Reporting of Observational Studies in Epidemiology Using Mendelian Randomization: The STROBE-MR Statement. *JAMA*. 2021;326(16):1614-1621. doi:10.1001/jama.2021.18236

13. Evangelou E, Warren HR, Mosen-Ansorena D, et al. Genetic analysis of over 1 million people identifies 535 new loci associated with blood pressure traits. *Nat Genet*. 2018;50(10):1412-1425. doi:10.1038/s41588-018-0205-x

14. Salam A, Atkins E, Sundström J, et al. Effects of blood pressure lowering on cardiovascular events, in the context of regression to the mean: A systematic review of randomized trials. *J Hypertens*. 2019;37(1):16-23. doi:10.1097/HJH.0000000000001994

15. Price AL, Patterson NJ, Plenge RM, Weinblatt ME, Shadick NA, Reich D. Principal components analysis corrects for stratification in genome-wide association studies. *Nat Genet*. 2006;38(8):904-909. doi:10.1038/ng1847

16. Garfield V, Salzmann A, Burgess S, Chaturvedi N. A Guide for Selection of Genetic Instruments in Mendelian randomisation (MR) studies of Type-2 diabetes and HbA1c: towards an integrated approach. *Diabetes*. 2023;72(2):175-183. doi:10.2337/db22-0110

17. Burgess S, Small DS, Thompson SG. A review of instrumental variable estimators for Mendelian randomization. *Stat Methods Med Res*. 2017;26(5):2333-2355. doi:10.1177/0962280215597579

18. Staplin N, Herrington WG, Murgia F, et al. Determining the Relationship Between Blood Pressure, Kidney Function, and Chronic Kidney Disease: Insights From Genetic Epidemiology. *Hypertension*. 2022;79(12):2671-2681. doi:10.1161/HYPERTENSIONAHA.122.19354

19. Hemani G, Tilling K, Smith GD. Orienting the causal relationship between imprecisely measured traits using GWAS summary data. *PLOS Genet*. 2017;13(11):e1007081. doi:10.1371/journal.pgen.1007081

20. Bowden J, Davey Smith G, Haycock PC, Burgess S. Consistent Estimation in Mendelian Randomization with Some Invalid Instruments Using a Weighted Median Estimator. *Genet Epidemiol*. 2016;40(4):304-314. doi:10.1002/gepi.21965

21. Bowden J, Davey Smith G, Burgess S. Mendelian randomization with invalid instruments: effect estimation and bias detection through Egger regression. *Int J Epidemiol*. 2015;44(2):512-525. doi:10.1093/ije/dyv080

22. Verbanck M, Chen CY, Neale B, Do R. Detection of widespread horizontal pleiotropy in causal relationships inferred from Mendelian randomization between complex traits and diseases. *Nat Genet*. 2018;50(5):693-698. doi:10.1038/s41588-018-0099-7

23. Lin DY, Tao R, Kalsbeek WD, et al. Genetic Association Analysis under Complex Survey Sampling: The Hispanic Community Health Study/Study of Latinos. *Am J Hum Genet*. 2014;95(6):675-688. doi:10.1016/j.ajhg.2014.11.005

Table S1. Number of deaths at ages 35-74 years by underlying cause (ICD-10 code) among 125 895 participants in the genetic analysis population

Underlying cause of death	ICD-10 codes (and number of deaths)*
**Vascular**	
Ischaemic heart disease (n=2005)	I200 (3), I209 (3), I210 (20), I211 (12), I213 (1), I219 (1775), I220 (2), I221 (1), I229 (1), I249 (32), I251 (48), I252 (1), I258 (10), I259 (96)
Cerebrovascular (n=746)	F019 (2), I600 (2), I602 (1), I608 (1), I609 (79), I61 (1), I610 (1), I612 (1), I613 (1), I614 (1), I615 (2), I618 (1), I619 (219), I620 (6), I629 (5), I633 (4), I634 (17), I635 (3), I638 (1), I639 (53), I64X (94), I669 (6), I671 (5), I672 (2), I673 (1), I674 (3), I678 (82), I679 (107), I690 (1), I691 (1), I693 (8), I694 (6), I698 (29)
Other vascular (n=727)	E115 (35), E145 (28), I011 (1), I018 (1), I050 (2), I051 (1), I059 (18), I069 (2), I070 (1), I071 (1), I079 (2), I080 (3), I081 (1), I091 (1), I099 (16), I110 (98), I119 (11), I260 (2), I269 (78), I270 (4), I272 (3), I279 (7), I319 (1), I330 (7), I340 (3), I350 (10), I351 (1), I358 (2), I38X (8), I420 (11), I421 (1), I426 (1), I429 (1), I442 (9), I443 (2), I460 (1), I469 (7), I471 (2), I472 (3), I489 (3), I48X (8), I490 (6), I499 (6), I500 (54), I501 (12), I509 (70), I515 (2), I517 (1), I518 (3), I519 (9), I710 (4), I712 (1), I713 (4), I718 (3), I719 (1), I729 (2), I731 (1), I739 (3), I740 (1), I741 (1), I743 (1), I771 (15), I776 (1), I779 (1), I802 (5), I803 (1), I822 (1), I828 (1), I829 (2), I830 (1), I839 (1), I872 (5), I879 (1), I890 (1), I99X (4), K550 (59), K551 (1), K552 (1), K559 (5), K761 (1), Q210 (1), Q213 (1), Q238 (1), Q248 (1), R570 (34)
**Kidney disease**	
Chronic kidney disease (n=1740)	E102 (10), E112 (862), E142 (336), I120 (108), I129 (1), I130 (3), I131 (2), I132 (27), N039 (25), N049 (2), N059 (11), N12X (12), N142 (1), N180 (11), N185 (32), N189 (225), N19X (65), N281 (1), N289 (5), Y841 (1)
Other kidney disease (n=313)	N002 (1), N009 (8), N10X (2), N151 (13), N179 (74), N200 (6), N201 (1), N300 (1), N309 (1), N390 (206)
**Other disease**	
Acute diabetic crisis (n=551)	E100 (3), E101 (2), E110 (164), E111 (182), E140 (105), E141 (88), E162 (7)
Infective[†] (n=816)	A047 (3), A060 (1), A090 (19), A099 (33), A09X (11), A170 (1), A181 (2), A182 (1), A183 (1), A199 (3), A415 (1), A419 (192), A480 (1), A483 (1), A498 (1), A810 (2), A86X (2), B200 (1), B201 (1), B207 (4), B208 (7), B210 (2), B212 (1), B218 (1), B227 (2), B238 (4), B24X (4), B259 (1), B373 (1), B451 (1), B462 (1), B465 (2), B690 (2), B948 (2), B99X (1), G009 (3), G039 (4), G042 (1), G049 (7), G060 (1), H440 (1), H664 (1), H669 (1), K052 (1), K223 (2), K228 (1), K251 (3), K254 (10), K255 (11), K256 (5), K259 (6), K264 (5), K265 (3), K269 (2), K274 (3), K275 (1), K290 (6), K291 (1), K292 (1), K295 (5), K297 (1), K318 (4), K352 (4), K353 (4), K358 (5), K359 (2), K37X (1), K572 (2), K578 (5), K579 (13), K610 (3), K611 (1), K612 (1), K650 (28), K658 (2), K659 (55), K920 (28), K921 (4), K922 (112), L021 (4), L022 (4), L023 (2), L024 (2), L031 (5), L038 (1), L039 (3), L089 (40), L899 (7), L89X (3), L984 (7), M009 (1), M600 (2), M725 (1), M726 (19), M798 (23), M869 (3), N410 (1), N419 (1), N498 (7), N719 (1), N739 (1), R572 (5)
Hepatobiliary (n=1070)	B169 (3), B171 (28), B182 (11), B189 (1), B190 (2), B199 (2), D136 (1), I850 (16), I859 (6), K701 (14), K702 (1), K703 (160), K704 (24), K709 (16), K711 (2), K716 (1), K720 (9), K721 (58), K729 (205), K739 (2), K742 (1), K743 (2), K745 (2), K746 (290), K750 (14), K754 (4), K759 (2), K764 (1), K766 (13), K767 (14), K768 (1), K769 (17), K800 (4), K801 (9), K802 (4), K803 (3), K804 (1), K805 (2), K810 (12), K811 (3), K819 (5), K822 (2), K829 (5), K830 (17), K831 (3), K839 (1), K851 (1), K852 (4), K858 (7), K859 (34), K85X (24), K861 (3), K868 (2), Q447 (1)
Neoplastic (n=2116)	C029 (12), C07X (2), C089 (1), C109 (3), C119 (1), C139 (1), C140 (5), C142 (1), C159 (16), C160 (2), C169 (201), C170 (10), C179 (1), C182 (3), C184 (1), C187 (2), C189 (112), C19X (4), C20X (21), C211 (1), C220 (51), C221 (23), C229 (105), C23X (25), C240 (12), C241 (9), C248 (3), C249 (14), C250 (22), C252 (1), C259 (89), C260 (3), C269 (2), C319 (1), C329 (12), C33X (1), C349 (141), C37X (1), C382 (1), C383 (1), C384 (1), C412 (2), C419 (7), C435 (1), C437 (1), C438 (1), C439 (10), C444 (1), C445 (1), C447 (1), C449 (7), C451 (2), C457 (1), C459 (4), C469 (1), C479 (1), C480 (8), C482 (4), C492 (4), C495 (1), C499 (14), C509 (231), C519 (3), C52X (1), C530 (1), C539 (118), C541 (24), C549 (2), C55X (11), C56X (102), C609 (2), C61X (75), C629 (1), C64X (84), C679 (23), C680 (2), C689 (1), C694 (1), C709 (2), C710 (13), C711 (1), C718 (2), C719 (42), C720 (1), C729 (1), C73X (27), C741 (1), C749 (1), C760 (6), C761 (1), C762 (3), C763 (2), C764 (1), C765 (2), C780 (7), C786 (5), C787 (7), C788 (2), C793 (3), C794 (3), C795 (1), C796 (1), C798 (3), C800 (27), C809 (19), C80X (2), C817 (1), C819 (9), C822 (1), C829 (1), C830 (1), C833 (13), C838 (1), C839 (3), C844 (1), C845 (3), C851 (1), C857 (2), C859 (44), C900 (42), C901 (1), C902 (1), C910 (20), C911 (2), C920 (26), C921 (7), C927 (2), C929 (3), C950 (2), C959 (2), C97X (1), D371 (6), D372 (1), D374 (6), D376 (6), D377 (2), D380 (2), D381 (6), D383 (2), D391 (4), D397 (1), D410 (3), D429 (1), D430 (13), D432 (1), D449 (1), D486 (1), D487 (9), D489 (3)
Respiratory[‡] (n=2072)	A162 (7), A165 (1), A169 (2), B206 (2), B440 (1), B441 (1), B909 (1), E848 (1), J069 (1), J09 (3), J09X (1), J100 (2), J110 (1), J111 (1), J129 (4), J151 (3), J157 (2), J159 (36), J180 (53), J181 (26), J182 (2), J189 (594), J209 (4), J22X (18), J348 (1), J391 (1), J42X (15), J439 (30), J440 (95), J441 (2), J448 (6), J449 (164), J459 (9), J46X (2), J47X (2), J60X (1), J64X (4), J677 (1), J679 (2), J680 (1), J690 (3), J80X (4), J81X (2), J841 (74),

	J848 (1), J849 (10), J850 (2), J852 (2), J869 (9), J90X (5), J939 (1), J942 (2), J960 (5), J961 (1), J969 (3), J980 (1), J981 (1), J984 (15), J985 (5), J988 (8), J989 (1), Q311 (1), U071 (513), U072 (299), U099 (1)
External/other/ill-defined (n=997)	D033 (1), D27X (1), D320 (1), D329 (9), D352 (1), D420 (1), D467 (1), D469 (6), D471 (1), D472 (1), D474 (1), D479 (1), D619 (4), D649 (5), D682 (1), D693 (2), D694 (1), D696 (3), D699 (3), D70X (2), D733 (1), D762 (1), E035 (1), E039 (12), E049 (1), E055 (1), E059 (3), E065 (1), E116 (3), E119 (8), E129 (1), E146 (3), E149 (5), E230 (1), E249 (2), E279 (1), E43X (1), E440 (1), E660 (1), E725 (1), E835 (1), E86X (4), E870 (1), E872 (10), E874 (1), E875 (2), E876 (1), E878 (1), E889 (2), F03X (6), F09X (1), F102 (5), F182 (1), F209 (2), G10X (6), G121 (1), G122 (17), G20X (7), G231 (1), G300 (1), G309 (2), G310 (1), G35X (1), G379 (1), G403 (1), G409 (10), G419 (2), G439 (1), G589 (1), G610 (5), G709 (1), G710 (2), G809 (1), G822 (1), G919 (4), G931 (10), G934 (5), G935 (1), G936 (2), G938 (1), G958 (1), I10X (3), K088 (1), K102 (1), K137 (1), K389 (1), K403 (1), K404 (1), K409 (1), K419 (1), K420 (4), K429 (2), K430 (2), K439 (2), K440 (1), K460 (3), K461 (1), K469 (2), K513 (1), K519 (1), K529 (6), K560 (2), K562 (1), K566 (40), K567 (1), K593 (5), K628 (1), K630 (1), K631 (23), K632 (4), K635 (1), K638 (1), K639 (5), K661 (1), K918 (1), K931 (1), L100 (1), L109 (1), L511 (2), L512 (1), L921 (1), L958 (1), L988 (1), M050 (1), M068 (1), M069 (16), M100 (1), M109 (1), M139 (1), M311 (2), M313 (1), M319 (1), M321 (3), M329 (1), M340 (1), M349 (1), M469 (1), M623 (5), M799 (1), N40X (8), N948 (1), O622 (1), O720 (1), R040 (1), R100 (3), R11X (1), R190 (1), R571 (17), R579 (2), R58X (3), R688 (20), R69X (1), R99X (190), S729 (2), T07X (1), T874 (1), V011 (1), V029 (1), V041 (1), V051 (1), V093 (3), V099 (64), V182 (1), V209 (1), V299 (1), V439 (1), V489 (1), V496 (1), V499 (12), V580 (1), V719 (1), V785 (1), V878 (6), V892 (10), V899 (3), W018 (1), W060 (1), W100 (13), W104 (1), W105 (1), W108 (2), W126 (1), W130 (9), W134 (2), W138 (1), W139 (2), W170 (4), W172 (1), W174 (1), W176 (1), W178 (2), W179 (1), W18 (1), W180 (5), W181 (1), W184 (2), W188 (1), W190 (8), W194 (1), W195 (1), W199 (3), W206 (1), W250 (1), W314 (1), W340 (1), W370 (1), W704 (1), W744 (2), W748 (3), W769 (1), W780 (1), W789 (1), W799 (1), W849 (1), W878 (2), X09 (1), X090 (3), X094 (1), X099 (1), X219 (1), X314 (1), X360 (1), X459 (1), X470 (1), X590 (3), X594 (1), X598 (1), X599 (28), X640 (1), X650 (1), X680 (1), X700 (5), X740 (3), X780 (3), X800 (1), X840 (1), X910 (1), X912 (1), X914 (2), X950 (3), X954 (17), X955 (1), X959 (1), X990 (6), X994 (5), X999 (2), Y044 (1), Y048 (1), Y084 (1), Y099 (1), Y159 (1), Y200 (1), Y240 (1), Y244 (3), Y245 (1), Y248 (1), Y249 (2), Y260 (2), Y280 (1), Y330 (1), Y334 (1), Y338 (1), Y340 (5), Y344 (6), Y346 (1), Y348 (2), Y349 (14), Y405 (1), Y579 (3), Y838 (1), Y839 (4), Y846 (1)

ICD-10: international classification of disease, 10th edition. * Only codes identified as primary causes of death in the study population are presented. † Excludes respiratory infections. ‡ Includes respiratory infections.

Table S2. Baseline characteristics of 125 895 participants (genetic analysis population) aged 35-74 years at recruitment by fifth of genetic risk score for diastolic blood pressure

	Genetic risk score for diastolic blood pressure					Difference (mean [SE] or %) between fifth V and fifth I
	I (n=25 179)	II (n=25 179)	III (n=25 179)	IV (n=25 179)	V (n=25 179)	
**Age, sex, ancestry and socioeconomic factors**						
Age (years)	50 (11)	50 (11)	50 (11)	50 (11)	50 (11)	-0.1 (0.1)
Men	33.1%	32.2%	31.8%	32.0%	32.1%	-1.0%
Indigenous American ancestry	65.4%	66.8%	66.9%	66.7%	65.1%	-0.3%
Resident in Coyoacán	38.7%	38.3%	38.2%	38.7%	39.0%	0.3%
University/high school education	16.6%	16.0%	15.7%	15.8%	16.2%	-0.5%
**Physical measurements**						
Systolic blood pressure (mmHg)	125.8 (16.3)	126.9 (16.7)	127.5 (17.1)	128.2 (17.1)	129.8 (17.9)	4.0 (0.2)
Diastolic blood pressure (mmHg)	82.0 (10.0)	83.0 (10.2)	83.3 (10.3)	83.9 (10.4)	85.1 (10.9)	3.1 (0.1)
Body mass index (kg/m^2)	29.1 (4.9)	29.2 (5.0)	29.2 (4.9)	29.1 (4.9)	29.2 (5.0)	0.1 (0.0)
Waist:hip ratio	0.90 (0.07)	0.90 (0.07)	0.90 (0.07)	0.90 (0.07)	0.90 (0.07)	0.00 (0.0)
**Lifestyle**						
Current smoker	52.4%	51.7%	51.2%	51.0%	52.1%	-0.3%
Current drinker	67.9%	68.0%	67.4%	67.6%	67.3%	-0.6%
Regular leisure time physical activity	22.4%	22.6%	22.3%	22.2%	22.7%	0.3%
**Medical history and medication use**						
Previously-diagnosed diabetes	13.6%	13.5%	13.1%	12.4%	12.1%	-1.5%
Previously-diagnosed hypertension	14.6%	17.6%	19.3%	21.1%	25.0%	10.4%
Antihypertensive medication use	10.6%	13.1%	14.0%	15.7%	18.9%	8.3%
Antithrombotic medication use	2.8%	2.6%	2.7%	2.8%	2.9%	0.1%
Lipid-lowering medication use	<1%	<1%	<1%	<1%	<1%	-0.1%

Mean (SD) or % shown. *for differences in means. SD: standard deviation, SE: standard error.

Table S3. Baseline characteristics of 133 027 participants (observational analysis population) aged 35-74 years at recruitment by baseline systolic blood pressure

	Baseline systolic blood pressure					
	**<115 mmHg (n=29 888)**	**115-124 mmHg (n=38 117)**	**125-134 mmHg (n=31 863)**	**135-144 mmHg (n=17 877)**	**≥145 mmHg (n=15 282)**	**Overall (n=133 027)**
**Age, sex, ancestry and socioeconomic factors**						
Age, years	46 (9)	48 (10)	51 (11)	55 (11)	58 (10)	50 (11)
Male	24.9%	33.4%	36.1%	35.5%	32.7%	32.4%
Indigenous American ancestry	66.6%	66.5%	66.3%	66.0%	65.8%	66.3%
Resident of Coyoacán	35.3%	38.3%	41.0%	42.3%	43.5%	39.4%
University/high school educated	20.5%	18.8%	14.7%	11.7%	8.7%	16.1%
**Physical measurements**						
Systolic blood pressure, mmHg	107.6 (5.9)	120.4 (2.5)	129.7 (2.6)	139.4 (2.6)	158.0 (12.5)	126.6 (16.1)
Diastolic blood pressure, mmHg	72.5 (6.6)	80.2 (5.1)	85.5 (5.9)	90.0 (6.9)	97.0 (10.2)	83.0 (10.1)
Body mass index, kg/m^2	27.5 (4.3)	28.7 (4.5)	29.8 (5.0)	30.3 (5.3)	30.5 (5.5)	29.1 (4.9)
Waist-hip ratio	0.88 (0.07)	0.89 (0.07)	0.91 (0.07)	0.91 (0.07)	0.92 (0.07)	0.90 (0.07)
**Lifestyle behaviours**						
Current smoker	36.5%	35.1%	32.0%	29.7%	25.1%	32.8%
Current drinker	69.8%	70.2%	68.6%	66.5%	61.1%	68.2%
Any regular leisure-time physical activity	22.5%	23.4%	22.3%	21.2%	20.3%	22.3%
**Medical history and medication use**						
Previously-diagnosed diabetes	7.3%	9.3%	13.6%	17.4%	24.0%	12.7%
Previously-diagnosed hypertension	5.0%	8.9%	19.5%	31.9%	51.5%	18.6%
Antihypertensive medication use	3.3%	6.0%	13.9%	23.2%	38.9%	13.4%
Anti-thrombotic medication use	1.7%	1.9%	2.4%	2.9%	3.8%	2.3%
Lipid-lowering medication use	<1%	<1%	<1%	<1%	<1%	<1%

Mean (SD) or % shown. Table excludes those with prior chronic disease at recruitment (ischaemic heart disease, stroke, chronic kidney disease, cirrhosis, cancer, emphysema), missing or extreme data on blood pressure or any covariate (sex, district of residence, educational level attained, anthropometry, smoking status, alcohol intake, leisure time physical activity, diabetes status). SD: standard deviation.

Table S4. Baseline characteristics of 133 027 participants (observational analysis population) aged 35-74 years at recruitment by baseline diastolic blood pressure

	Baseline diastolic blood pressure					
	**≤75 mmHg (n=26 879)**	**76-80 mmHg (n=28 237)**	**81-85 mmHg (n=27 017)**	**86-90 mmHg (n=25 371)**	**>90 mmHg (n=25 523)**	**Overall (n=133 027)**
**Age, sex, ancestry and socioeconomic factors**						
Age, years	47 (10)	49 (10)	50 (10)	52 (11)	54 (10)	50 (11)
Male	23.8%	31.4%	34.1%	35.6%	37.3%	32.4%
Indigenous American ancestry	66.7%	66.6%	66.2%	66.3%	65.1%	66.3%
Resident of Coyoacán	35.5%	36.0%	39.7%	41.4%	45.2%	39.4%
University/high school educated	18.9%	17.5%	17.0%	14.2%	12.5%	16.1%
**Physical measurements**						
Systolic blood pressure, mmHg	110.7 (10.2)	120.3 (9.1)	125.0 (9.0)	132.7 (10.2)	146.0 (15.6)	126.6 (16.1)
Diastolic blood pressure, mmHg	69.4 (4.3)	78.7 (1.6)	82.4 (1.3)	88.1 (1.7)	97.5 (7.0)	83.0 (10.1)
Body mass index, kg/m²	27.6 (4.4)	28.6 (4.6)	29.2 (4.7)	29.9 (5.1)	30.6 (5.3)	29.1 (4.9)
Waist-hip ratio	0.88 (0.07)	0.89 (0.07)	0.90 (0.07)	0.91 (0.07)	0.92 (0.07)	0.90 (0.07)
**Lifestyle behaviours**						
Current smoker	35.4%	34.0%	33.3%	31.3%	29.7%	32.8%
Current drinker	68.6%	68.6%	69.2%	67.8%	66.4%	68.2%
Any regular leisure-time physical activity	22.2%	23.3%	22.7%	22%.0	21.1%	22.3%
**Medical history and medication use**						
Previously-diagnosed diabetes	9.7%	11.0%	11.8%	14.4%	16.8%	12.7%
Previously-diagnosed hypertension	6.8%	10.8%	13.8%	23.5%	39.6%	18.6%
Antihypertensive medication use	4.9%	7.5%	9.6%	17.1%	29.2%	13.4%
Anti-thrombotic medication use	1.9%	1.9%	2.1%	2.6%	3.1%	2.3%
Lipid-lowering medication use	<1%	<1%	<1%	<1%	<1%	<1%

Mean (SD) or % shown. Table excludes those with prior chronic disease at recruitment (ischaemic heart disease, stroke, chronic kidney disease, cirrhosis, cancer, emphysema), missing or extreme data on blood pressure or any covariate (sex, district of residence, educational level attained, anthropometry, smoking status, alcohol intake, leisure time physical activity, diabetes status). SD: standard deviation.

Table S5. Genetic associations of SBP with cause-specific mortality using robust Mendelian randomization methods based on summary-level data

Cause of death	Mortality RR (95% CI) per 10mmHg higher genetically-predicted SBP				
	IVW *	Weighted median †	MR-Egger ‡	MR Egger intercept	MR PRESSO §
Ischemic heart disease	1.70 (1.46 - 1.97)	1.60 (1.24 - 2.06)	1.64 (1.29 - 2.10)	0.0050	1.70 (1.46 - 1.97)
Cerebrovascular disease	1.79 (1.40 - 2.28)	2.82 (1.83 - 4.32)	2.32 (1.55 - 3.48)	-0.0415	1.55 (1.13 - 2.11)
Other vascular disease	1.42 (1.11 - 1.82)	1.94 (1.25 - 3.01)	1.37 (0.91 - 2.07)	0.0052	1.42 (1.10 - 1.83)
**All vascular disease**	**1.65 (1.48 - 1.85)**	**1.54 (1.27 - 1.87)**	**1.71 (1.42 - 2.06)**	**-0.0051**	**1.65 (1.47 - 1.86)**
Chronic kidney disease	1.41 (1.20 - 1.65)	1.29 (0.97 - 1.71)	1.28 (0.98 - 1.67)	0.0150	1.41 (1.18 - 1.67)
Other kidney disease	1.27 (0.87 - 1.85)	1.11 (0.58 - 2.12)	1.15 (0.62 - 2.16)	0.0150	1.27 (0.87 - 1.85)
**Any kidney disease**	**1.38 (1.19 - 1.60)**	**1.26 (0.97 - 1.63)**	**1.26 (0.98 - 1.61)**	**0.0150**	**1.38 (1.18 - 1.62)**
Acute diabetic crises	1.10 (0.83 - 1.47)	1.01 (0.62 - 1.64)	0.93 (0.58 - 1.49)	0.0274	1.10 (0.83 - 1.46)
Hepatobiliary disease	0.90 (0.73 - 1.10)	0.84 (0.59 - 1.20)	0.7 (0.5 - 0.98)	0.0393	0.90 (0.73 - 1.10)
Infection	1.03 (0.81 - 1.30)	0.82 (0.54 - 1.23)	1.03 (0.7 - 1.51)	-0.0002	1.03 (0.81 - 1.30)
Cancer	0.96 (0.83 - 1.10)	1.04 (0.81 - 1.32)	0.89 (0.7 - 1.13)	0.0109	0.96 (0.83 - 1.10)
Respiratory disease	1.04 (0.90 - 1.20)	1.11 (0.86 - 1.44)	1.02 (0.8 - 1.3)	0.0036	1.04 (0.90 - 1.20)
External/other/ill-defined	1.31 (1.06 - 1.61)	1.26 (0.88 - 1.82)	1.06 (0.75 - 1.51)	0.0325	1.31 (1.06 - 1.60)
**All-cause**	**1.22 (1.15 - 1.30)**	**1.19 (1.07 - 1.32)**	**1.15 (1.04 - 1.26)**	**0.0103**	**1.22 (1.15 - 1.30)**

All analyses are adjusted for age, age-squared, body mass index, district of residence and seven genetic principal components. For each approach the SNP-SBP effect estimates and their standard errors were taken from the Million Veteran Program trans-ancestry meta-analysis GWAS of mean SBP, with the alleles aligned to be SBP-increasing.

* An IVW linear regression of the 1953 SNP-mortality effects on the 1953 (aligned) SNP-SBP effects is performed in which the regression line is forced through the origin. It relies on the assumption of no 'horizontal pleiotropy' (ie, it assumes that all of the SNPs affect mortality only through SBP and not through any other mechanism).

† The weighted median approach applies the ratio method to each SNP, orders the results, assigns them normalised inverse-variance weights (to capture the 'information content' of each SNP), then selects the ratio estimate at which the cumulative information content first passes half of the total information content. It relies on the assumption that at least half of the statistical information on the SNP-SBP associations comes from valid instrumental variables. It is more robust to individual genetic variants with strongly outlying causal estimates compared with the IVW and MR-Egger methods

‡ A modifications of the IVW approach in which the regression is not forced through the origin. The intercept provides an estimate of net horizontal pleiotropic effects under the assumption that any such effects are uncorrelated with the SNP-exposure effects (known as the InSIDE assumption). For each RR shown in the above table, the intercept term was non-significant at the 5% level after correction for multiple testing.

§ Mendelian Randomization Pleiotropy RESidual Sum and Outlier (MR-PRESSO) identifies SNPs with horizontal pleiotropic effects based on their contributions to heterogeneity and removes them from the analyses. A single outlier was found for the chronic kidney disease and two for all-cause mortality, and none for the other analyses. CI: confidence interval, IVW: inverse variance weighted, MR: Mendelian randomization, RR: rate ratio, SBP: systolic blood pressure, SNP: single nucleotide polymorphism.

Table S6. Genetic associations of DBP with cause-specific mortality using robust Mendelian randomization methods based on summary-level data

Cause of death	Mortality RR (95% CI) per 5mmHg higher genetically-predicted DBP				
	IVW *	Weighted median †	MR-Egger ‡	MR Egger intercept	MR PRESSO §
Ischemic heart disease	1.22 (1.08 - 1.38)	1.22 (1.00 - 1.50)	1.13 (0.93 - 1.38)	0.0074	1.22 (1.07 - 1.38)
Cerebrovascular disease	1.43 (1.17 - 1.75)	1.34 (0.96 - 1.88)	1.37 (0.99 - 1.90)	0.0040	1.35 (1.04 - 1.75)
Other vascular disease	1.37 (1.11 - 1.68)	1.65 (1.18 - 2.30)	1.53 (1.10 - 2.12)	-0.0107	1.37 (1.11 - 1.69)
**All vascular disease**	**1.29 (1.17 - 1.42)**	**1.29 (1.10 - 1.51)**	**1.25 (1.08 - 1.46)**	**0.0029**	**1.29 (1.17 - 1.43)**
Chronic kidney disease	0.91 (0.79 - 1.04)	1.06 (0.84 - 1.33)	0.87 (0.70 - 1.08)	0.0040	0.90 (0.78 - 1.04)
Other kidney disease	1.03 (0.75 - 1.42)	0.79 (0.47 - 1.32)	0.83 (0.50 - 1.37)	0.0213	1.03 (0.75 - 1.42)
**Any kidney disease**	**0.93 (0.82 - 1.05)**	**0.98 (0.79 - 1.20)**	**0.86 (0.71 - 1.05)**	**0.0063**	**0.93 (0.81 - 1.06)**
Acute diabetic crises	0.78 (0.61 - 0.99)	0.68 (0.46 - 1.01)	0.66 (0.45 - 0.97)	0.0159	0.78 (0.61 - 0.99)
Hepatobiliary disease	1.18 (0.99 - 1.40)	1.38 (1.04 - 1.83)	1.43 (1.09 - 1.89)	-0.0188	1.18 (0.99 - 1.40)
Infection	1.01 (0.83 - 1.23)	0.88 (0.64 - 1.22)	1.05 (0.77 - 1.43)	-0.0035	1.01 (0.83 - 1.23)
Cancer	0.99 (0.88 - 1.12)	1.06 (0.87 - 1.30)	1.03 (0.85 - 1.25)	-0.0044	0.99 (0.88 - 1.11)
Respiratory disease	0.98 (0.86 - 1.10)	0.99 (0.81 - 1.21)	1.03 (0.84 - 1.25)	-0.0050	0.98 (0.86 - 1.10)
External/other/ill-defined	1.19 (1.00 - 1.42)	1.18 (0.89 - 1.58)	1.19 (0.90 - 1.58)	-0.0001	1.19 (1.00 - 1.42)
**All-cause**	**1.07 (1.02 - 1.12)**	**1.05 (0.97 - 1.14)**	**1.08 (1.00 - 1.16)**	**-0.0007**	**1.08 (1.02 - 1.13)**

All analyses are adjusted for age, age-squared, body mass index, district of residence and seven genetic principal components. For each approach the SNP-DBP effect estimates and their standard errors were taken from the Million Veteran Program trans-ancestry meta-analysis GWAS of mean DBP, with the alleles aligned to be DBP-increasing.

* An IVW linear regression of the 1953 SNP-mortality effects on the 1953 (aligned) SNP-DBP effects is performed in which the regression line is forced through the origin. It relies on the assumption of no 'horizontal pleiotropy' (ie, it assumed that all of the SNPs affect mortality only through DBP and not through any other mechanism).

† The weighted median approach applies the ratio method to each SNP, orders the results, assigns them normalised inverse-variance weights (to capture the 'information content' of each SNP), then selects the ratio estimate at which the cumulative information content first passes half of the total information content. It relies on the assumption that at least half of the statistical information on the SNP-DBP associations comes from valid instrumental variables. It is more robust to individual genetic variants with strongly outlying causal estimates compared with the IVW and MR-Egger methods

‡ A modifications of the IVW approach in which the regression is not forced through the origin. The intercept provides an estimate of net horizontal pleiotropic effects under the assumption that any such effects are uncorrelated with the SNP-exposure effects (known as the InSIDE assumption). For each RR shown in the above table, the intercept term was non-significant at the 5% level after correction for multiple testing.

§ Mendelian Randomization Pleiotropy RESidual Sum and Outlier (MR-PRESSO) identifies SNPs with horizontal pleiotropic effects based on their contributions to heterogeneity and removes them from the analyses. A single outlier was found for the chronic kidney disease and two for all-cause mortality, and none for the other analyses. CI: confidence interval, DBP: diastolic blood pressure, IVW: inverse variance weighted, MR: Mendelian randomization, RR: rate ratio, SNP: single nucleotide polymorphism.

Figure S1: Participant flow diagram

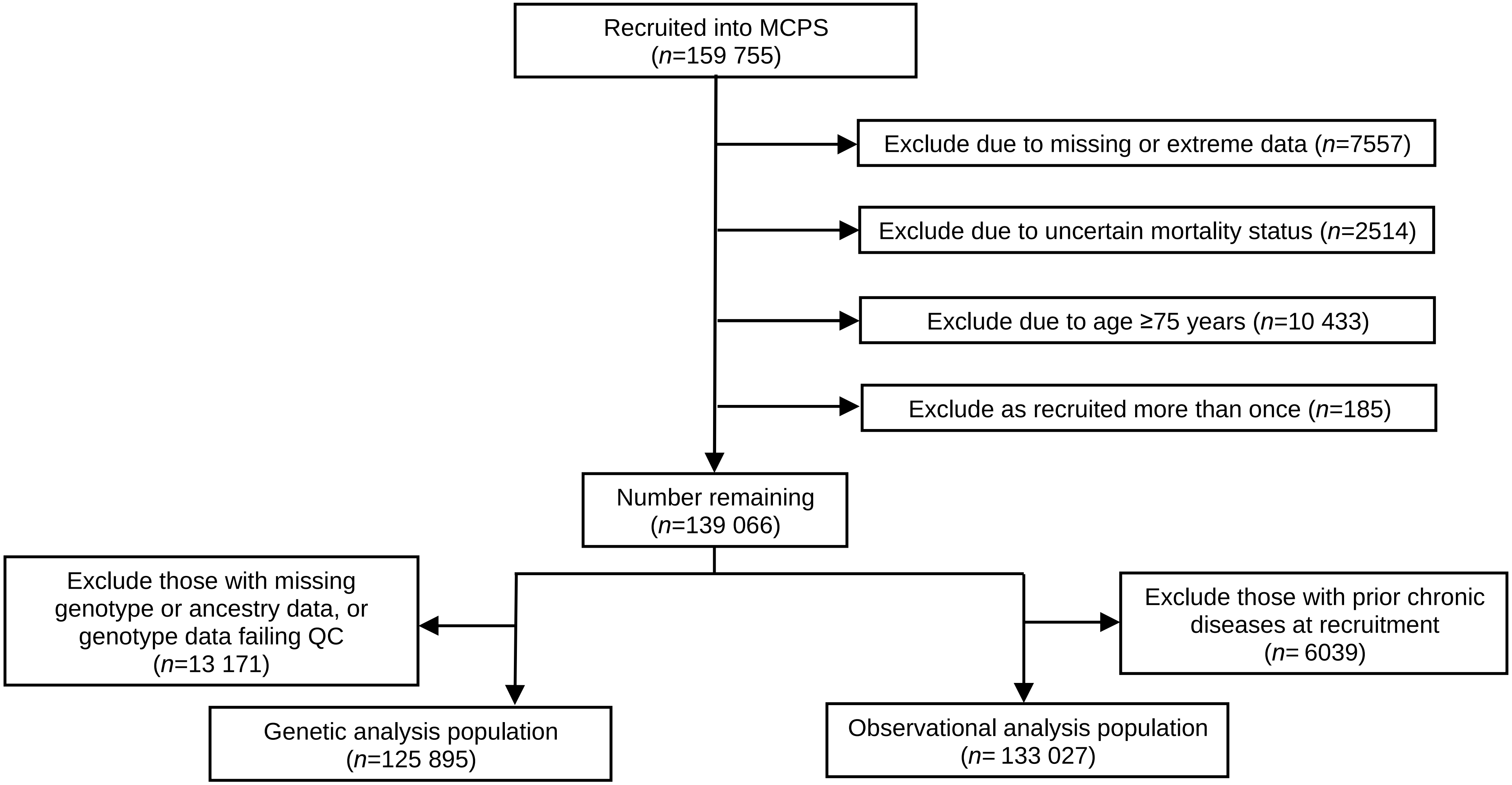

Figure S2: Comparison of effect sizes for genetic variants in SBP−GRS and DBP−GRS

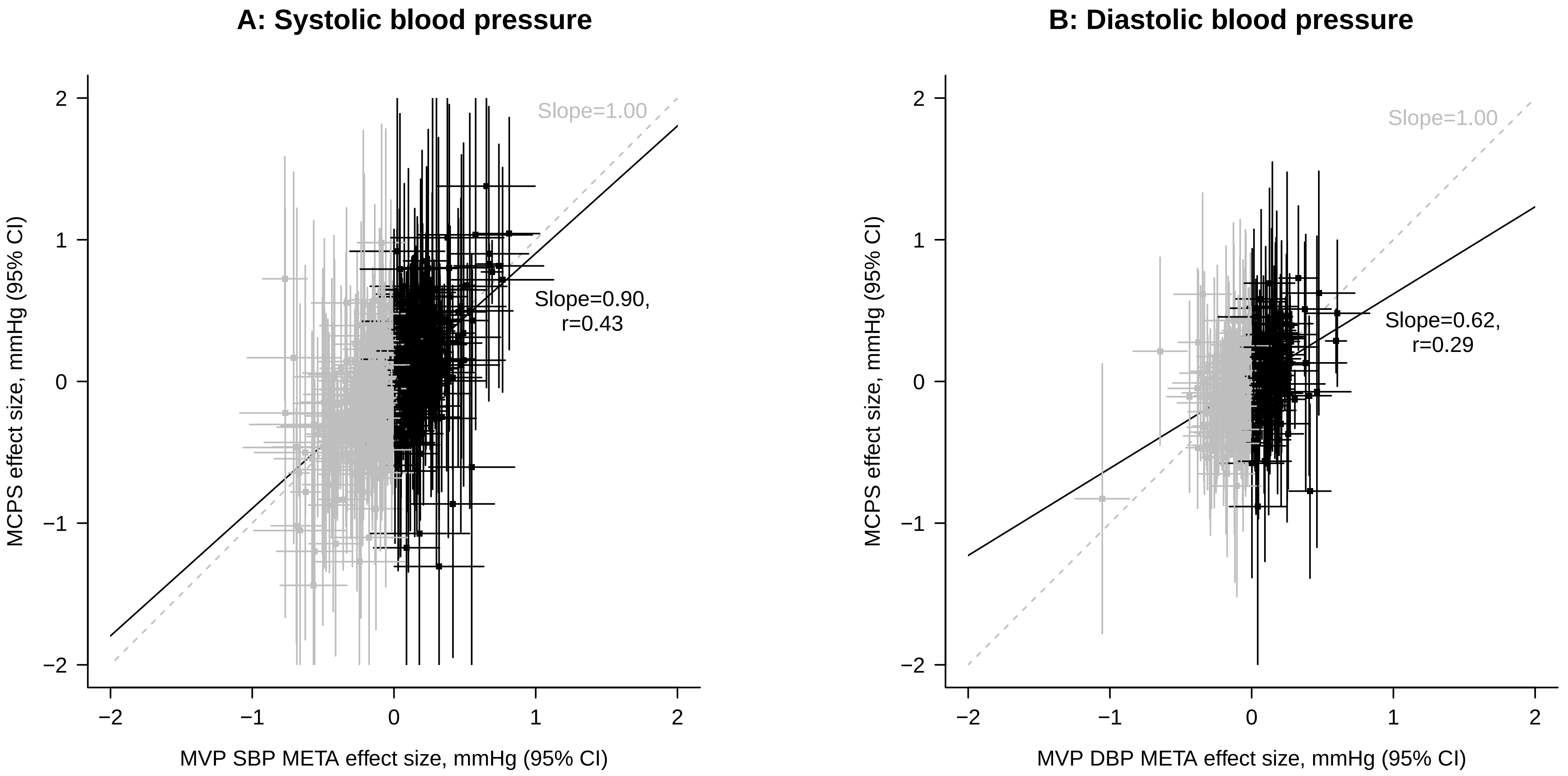

Shown are 1953 variants from the SBP (A) and DBP (B) genetic scores with their effect sizes from the MVP and MCPS GWAS. To aid interpretation, effect sizes are displayed as listed in the MVP results. Variants with negative effect estimates in MVP (coloured grey here) are re−orientated so the trait−increasing allele is used when GRS are calculated. CI: confidence interval, DBP: diastolic blood pressure, MCPS: Mexico City Prospective Study, MVP: Million Veteran Program, SBP: systolic blood pressure

Figure S3: Association of SBP GRS with SBP at recruitment by age, district and Indigenous American ancestry proportion

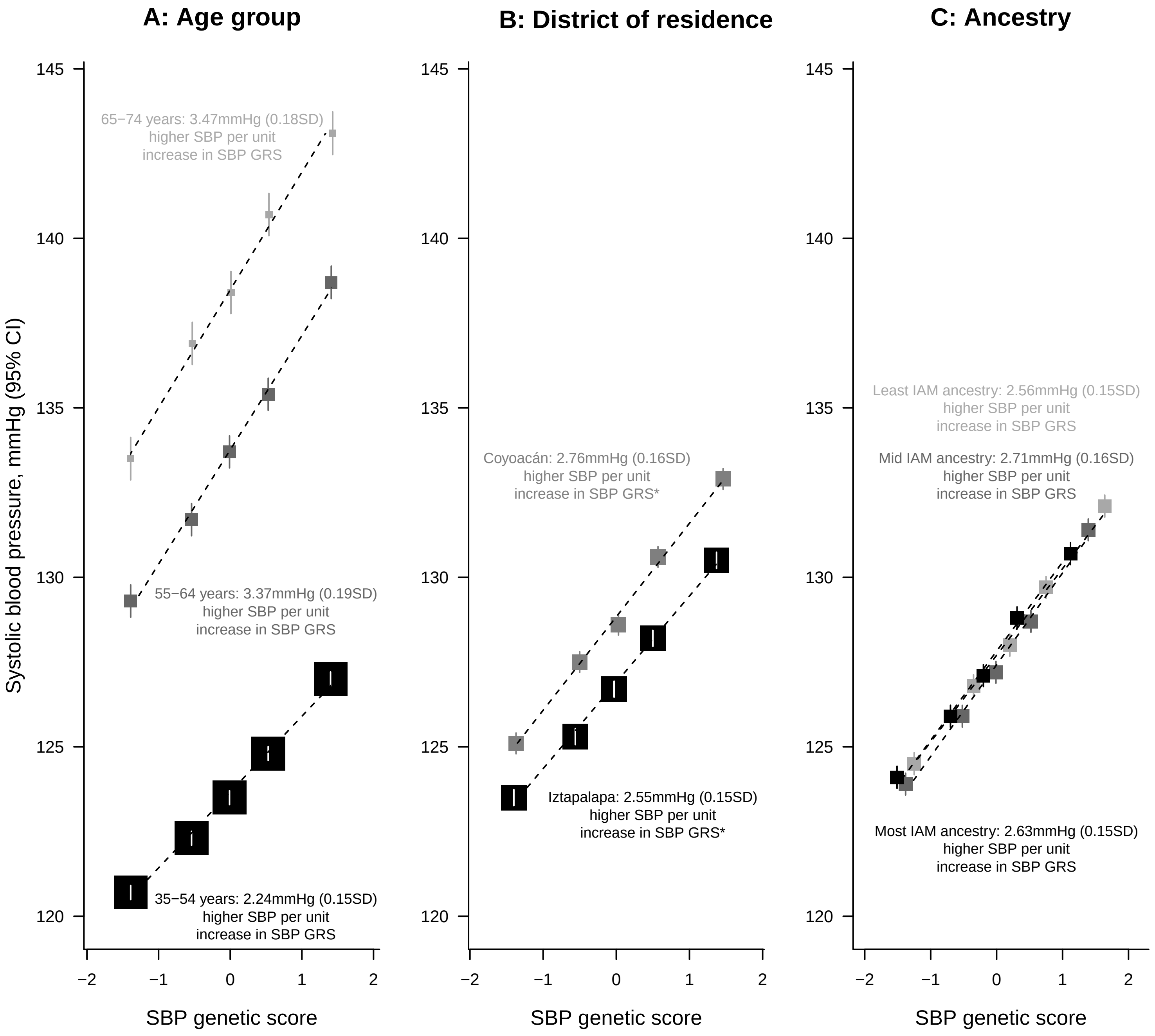

Each panel shows five equally−sized groups for each cohort stratum. *Adjusted for age, age−squared, sex, BMI and seven genetic PCs. All other estimates are adjusted for age, age−squared, sex, BMI, district of residence and seven genetic PCs. BMI: body mass index, CI: confidence interval, DBP: diastolic blood pressure, GRS: genetic risk score, IAM: Indigenous American ancestry, PC: principal component, RR: rate ratio, SBP: systolic blood pressure, SD: standard deviation

Figure S4: Association of DBP GRS with DBP at recruitment by age, district and Indigenous American ancestry proportion

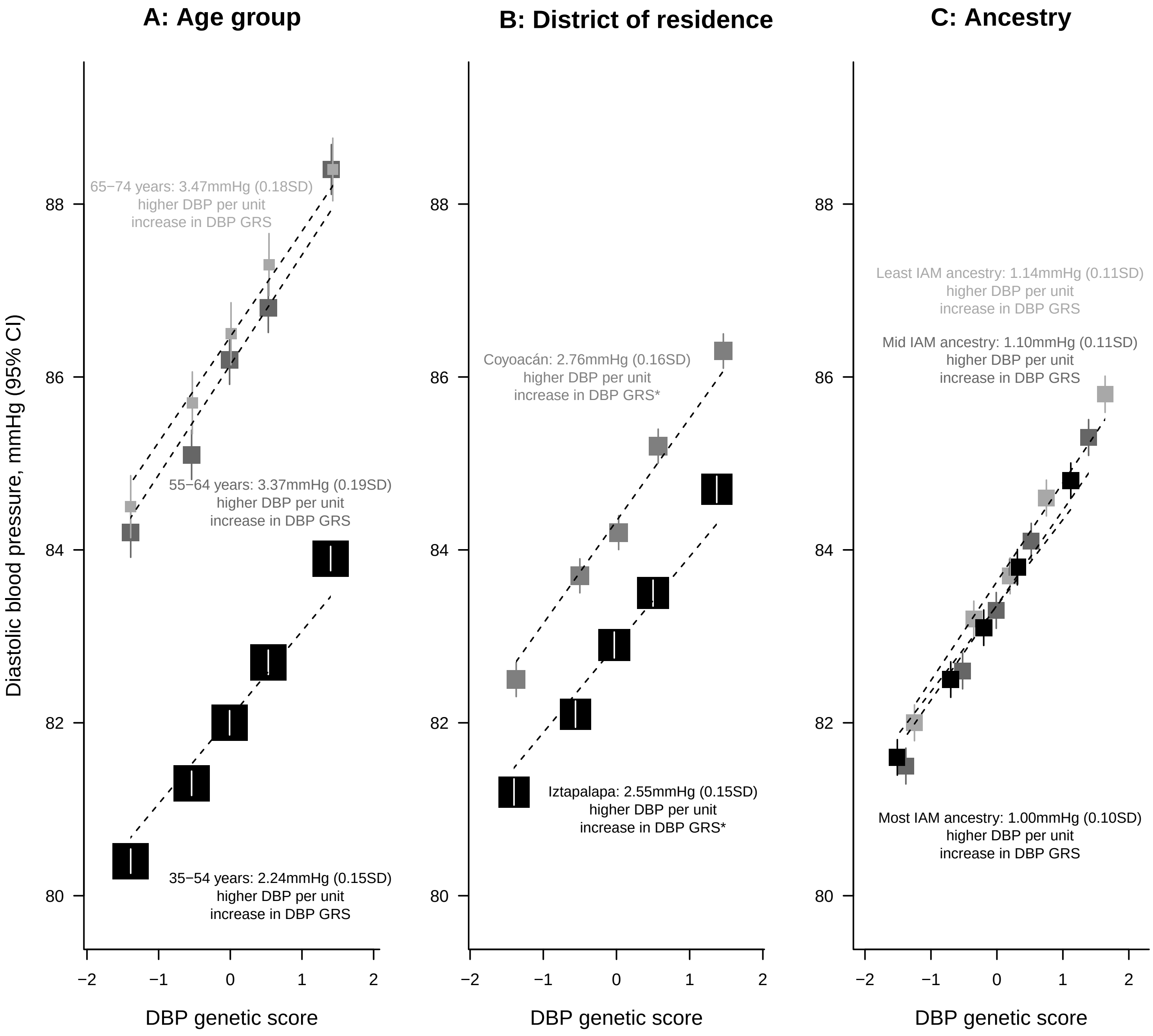

Each panel shows five equally−sized groups for each cohort stratum. *Adjusted for age, age−squared, sex, BMI and seven genetic PCs. All other estimates are adjusted for age, age−squared, sex, BMI, district of residence and seven genetic PCs. BMI: body mass index, CI: confidence interval, DBP: diastolic blood pressure, GRS: genetic risk score, IAM: Indigenous American ancestry, PC: principal component, RR: rate ratio, SBP: systolic blood pressure, SD: standard deviation

Figure S5: Associations between genetically-predicted DBP and cardiovascular and kidney mortality by diabetes status

Cause of death	No. of deaths	RR (95% CI)	Heterogeneity test by diabetes
Ischemic heart disease			
Diabetes	771	1.20 (0.70, 1.95)	χ^2=1.2
No diabetes	1234	1.58 (1.09, 2.22)	p=0.28
Cerebrovascular disease			
Diabetes	259	1.65 (0.86, 2.94)	χ^2=0.084
No diabetes	487	1.80 (1.12, 2.78)	p=0.77
Other vascular disease			
Diabetes	216	1.35 (0.68, 2.48)	χ^2=0.93
No diabetes	511	1.81 (1.13, 2.78)	p=0.33
All vascular disease			
Diabetes	1246	1.31 (0.78, 2.09)	χ^2=1.1
No diabetes	2232	1.67 (1.21, 2.27)	p=0.30
Any kidney disease			
Diabetes	1394	0.88 (0.53, 1.40)	χ^2=2.9
No diabetes	659	1.29 (0.82, 1.95)	p=0.087

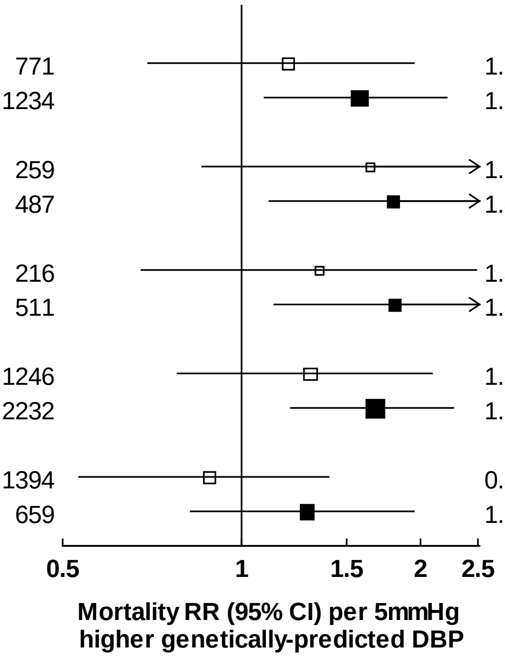

Mortality rate ratios (RRs) are per 5mmHg higher genetically-predicted DBP and are stratified by diabetes status and adjusted for age-at-risk, sex, body mass index and the first 7 genetic principal components. The size of each square is inversely proportional to the variance of the log RR. Horizontal lines represent 95% confidence intervals (CI). DBP = diastolic blood pressure.

Figure S6: Comparison of Steiger-filtered and primary SBP analyses

Cause of death	A: Steiger-filtered No. of deaths	A: Steiger-filtered RR (95% CI)	B: Primary Analysis No. of deaths	B: Primary Analysis RR (95% CI)
**Vascular diseases**				
Ischemic heart disease	2005	1.80 (1.42, 2.27)	2005	1.78 (1.44, 2.19)
Cerebrovascular disease	746	1.99 (1.44, 2.72)	746	1.88 (1.40, 2.51)
Other vascular disease	727	1.52 (1.09, 2.09)	727	1.45 (1.07, 1.95)
**All vascular disease**	**3478**	**1.78 (1.44, 2.18)**	**3478**	**1.73 (1.44, 2.06)**
**Kidney disease**				
Chronic kidney disease	1740	1.50 (1.17, 1.90)	1740	1.45 (1.16, 1.80)
Other kidney disease	313	1.31 (0.82, 2.07)	313	1.29 (0.83, 1.98)
**Any kidney disease**	**2053**	**1.47 (1.16, 1.85)**	**2053**	**1.42 (1.15, 1.75)**
**Other diseases**				
Acute diabetic crisis	551	1.16 (0.79, 1.66)	551	1.12 (0.79, 1.56)
Hepatobiliary disease	1070	0.87 (0.65, 1.15)	1070	0.88 (0.67, 1.14)
Infection*	816	1.02 (0.74, 1.39)	816	1.02 (0.76, 1.36)
Cancer	2116	0.94 (0.75, 1.18)	2116	0.95 (0.77, 1.16)
Respiratory disease**	2072	1.04 (0.82, 1.30)	2072	1.04 (0.84, 1.28)
External/other/ill-defined	997	1.42 (1.06, 1.89)	997	1.34 (1.02, 1.74)
**All-cause mortality**	**13153**	**1.26 (1.06, 1.49)**	**13153**	**1.24 (1.08, 1.43)**

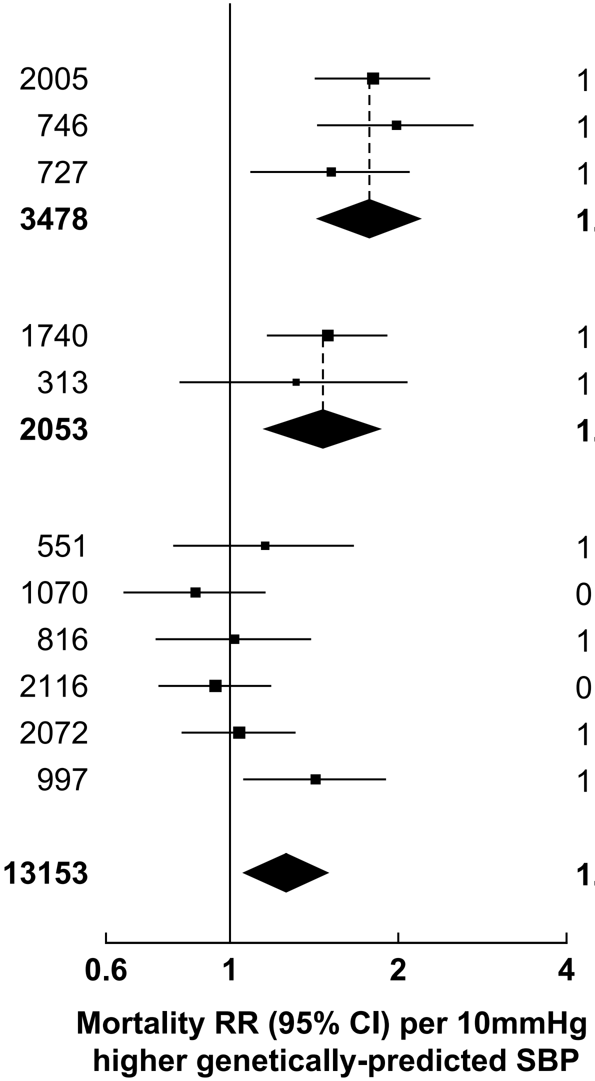

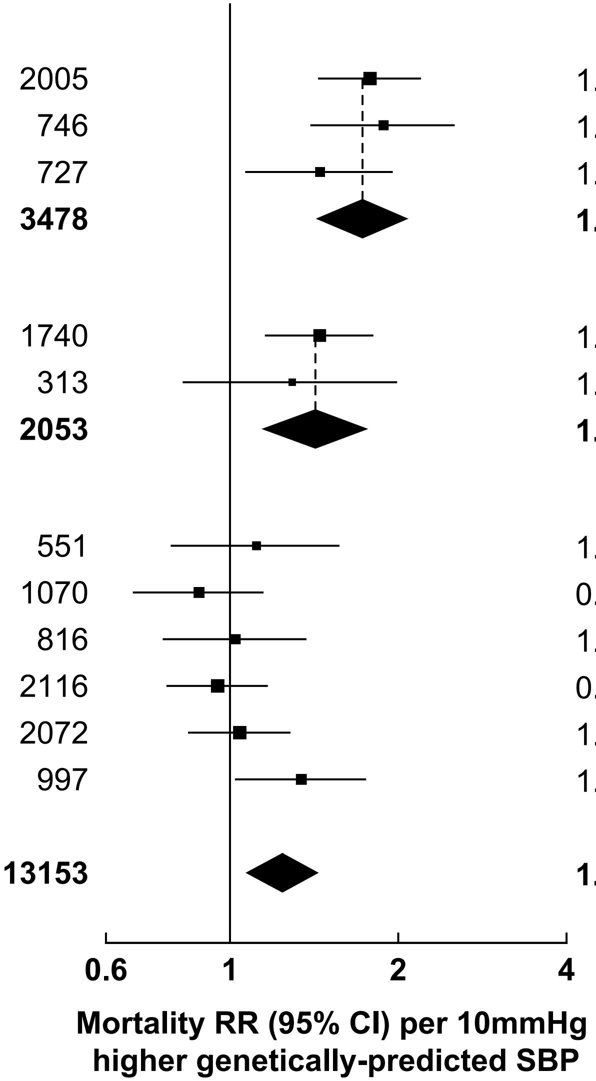

Mortality rate ratios (RRs) are per 10mmHg higher genetically-predicted SBP using a Steiger-filtered genetic score for SBP from which variants with a significantly stronger effect on estimated glomerular filtration rate are removed (A), and the primary analyses (B). Both sets of analyses are adjusted for age-at-risk, sex, body mass index, district of residence and the first 7 genetic principal components. The size of each square is inversely proportional to the variance of the log RR. Horizontal lines represent 95% confidence intervals (CI). SBP: systolic blood pressure.

Figure S7: Comparison of Steiger-filtered and primary DBP analyses

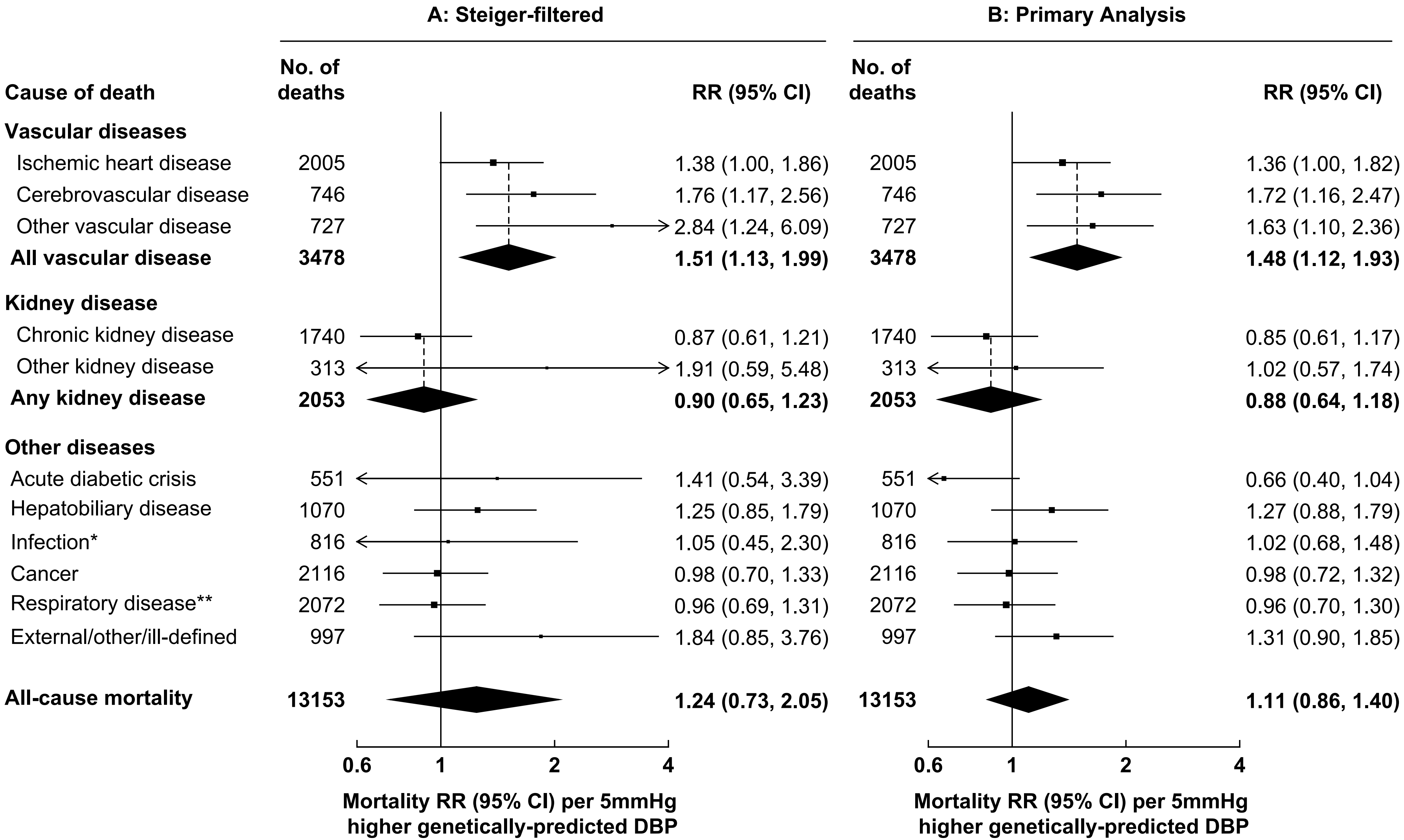

Mortality rate ratios (RRs) are per 5mmHg higher genetically-predicted DBP using a Steiger-filtered genetic score for SBP from which variants with a significantly stronger effect on estimated glomerular filtration rate are removed (A), and the primary analyses (B). Both sets of analyses are adjusted for age-at-risk, sex, body mass index, district of residence and the first 7 genetic principal components. The size of each square is inversely proportional to the variance of the log RR. Horizontal lines represent 95% confidence intervals (CI). DBP: diastolic blood pressure.

Figure S8: Associations between genetically-predicted SBP and cardiovascular and kidney mortality by age group

Cause of death	No. of deaths	RR (95% CI)	Heterogeneity test by age
Ischemic heart disease			
35-54 years	216	1.88 (1.01, 3.39)	χ^2=3.7,
55-64 years	618	1.79 (1.31, 2.44)	p=0.16
65-74 years	1259	1.42 (1.05, 1.91)	
Cerebrovascular disease			
35-54 years	98	2.19 (0.89, 5.00)	χ^2=2.7,
55-64 years	232	1.73 (1.12, 2.63)	p=0.26
65-74 years	450	1.52 (1.05, 2.18)	
Other vascular disease			
35-54 years	91	1.24 (0.46, 3.05)	χ^2=0.65,
55-64 years	235	1.34 (0.86, 2.03)	p=0.72
65-74 years	429	1.36 (0.94, 1.95)	
All vascular disease			
35-54 years	405	1.78 (1.11, 2.80)	χ^2=2.8,
55-64 years	1085	1.67 (1.27, 2.19)	p=0.25
65-74 years	2138	1.43 (1.08, 1.89)	
Any kidney disease			
35-54 years	334	1.56 (0.92, 2.57)	χ^2=1.5,
55-64 years	746	1.28 (0.95, 1.71)	p=0.48
65-74 years	1059	1.28 (0.95, 1.73)	

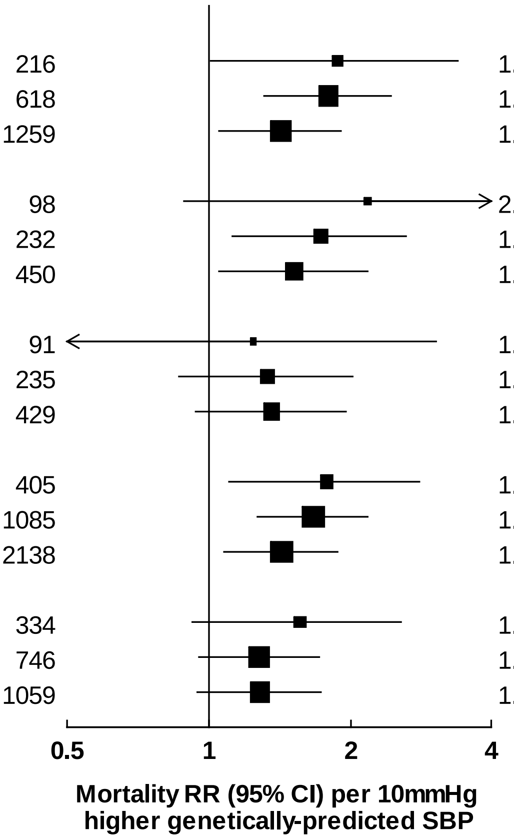

Mortality rate ratios (RRs) are per 10mmHg higher genetically-predicted SBP and are stratified by age-at-risk and adjusted for sex, body mass index, district of residence and the first 7 genetic principal components. The size of each square is inversely proportional to the variance of the log RR. Horizontal lines represent 95% confidence intervals (CI). SBP = systolic blood pressure.

Figure S9: Associations between genetically-predicted DBP and cardiovascular and kidney mortality by age group

Cause of death	No. of deaths	RR (95% CI)	Heterogeneity test by age
Ischemic heart disease			
35-54 years	216	1.65 (0.79, 3.14)	χ^2=0.65,
55-64 years	618	1.25 (0.74, 2.01)	p=0.72
65-74 years	1259	1.30 (0.70, 2.27)	
Cerebrovascular disease			
35-54 years	98	1.58 (0.54, 3.80)	χ^2=2.6,
55-64 years	232	1.23 (0.63, 2.23)	p=0.27
65-74 years	450	1.80 (0.90, 3.31)	
Other vascular disease			
35-54 years	91	0.96 (0.28, 2.58)	χ^2=3.9,
55-64 years	235	1.70 (0.89, 3.02)	p=0.14
65-74 years	429	1.84 (0.91, 3.39)	
All vascular disease			
35-54 years	405	1.45 (0.81, 2.45)	χ^2=0.36,
55-64 years	1085	1.33 (0.83, 2.07)	p=0.84
65-74 years	2138	1.49 (0.81, 2.57)	
Any kidney disease			
35-54 years	334	0.89 (0.45, 1.63)	χ^2=0.21,
55-64 years	746	0.83 (0.49, 1.32)	p=0.90
65-74 years	1059	0.96 (0.51, 1.68)	

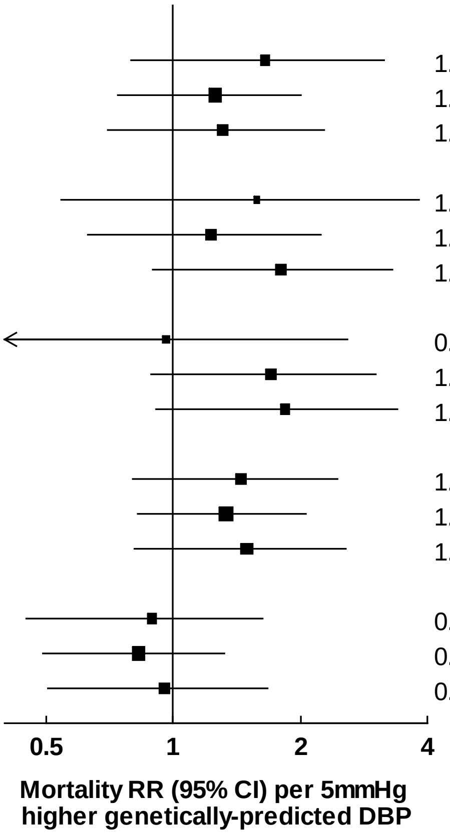

Mortality rate ratios (RRs) are per 5mmHg higher genetically-predicted DBP and are stratified by age-at-risk and adjusted for sex, body mass index, district of residence and the first 7 genetic principal components. The size of each square is inversely proportional to the variance of the log RR. Horizontal lines represent 95% confidence intervals (CI). DBP = diastolic blood pressure.

Figure S10: Associations between genetically-predicted SBP and cardiovascular and kidney mortality by biological sex

Cause of death	No. of deaths	RR (95% CI)	Heterogeneity test by sex
Ischemic heart disease			
Men	1035	1.66 (1.18, 2.30)	χ^2=0.74,
Women	970	1.95 (1.47, 2.56)	p=0.39
Cerebrovascular disease			
Men	300	1.98 (1.20, 3.20)	χ^2=0.11,
Women	446	1.82 (1.25, 2.62)	p=0.74
Other vascular disease			
Men	276	1.16 (0.68, 1.92)	χ^2=2.0,
Women	451	1.65 (1.13, 2.37)	p=0.16
All vascular disease			
Men	1611	1.61 (1.18, 2.17)	χ^2=0.63,
Women	1867	1.84 (1.46, 2.31)	p=0.43
Any kidney disease			
Men	828	1.57 (1.09, 2.22)	χ^2=0.64,
Women	1225	1.34 (1.03, 1.73)	p=0.42

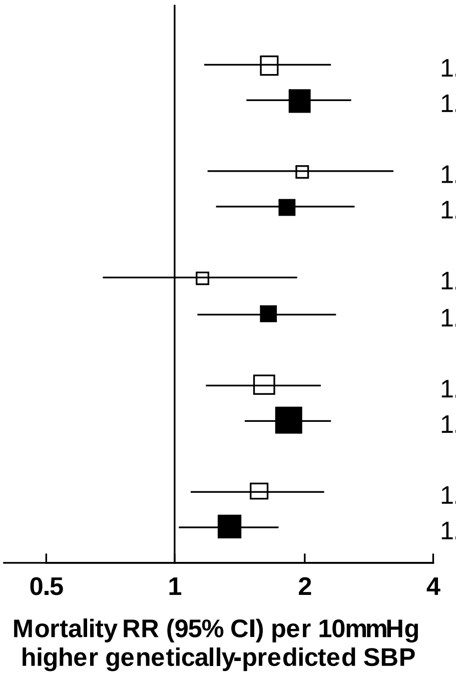

Mortality rate ratios (RRs) are per 10mmHg higher genetically-predicted SBP and are stratified by sex and adjusted for age-at-risk, body mass index, district of residence and the first 7 genetic principal components. The size of each square is inversely proportional to the variance of the log RR. Horizontal lines represent 95% confidence intervals (CI). SBP = systolic blood pressure.

Figure S11: Associations between genetically-predicted DBP and cardiovascular and kidney mortality by biological sex

Cause of death	No. of deaths	RR (95% CI)	Heterogeneity test by sex
Ischemic heart disease			
Men	1035	1.38 (0.80, 2.25)	χ^2=0.0044,
Women	970	1.36 (0.91, 1.95)	p=0.95
Cerebrovascular disease			
Men	300	1.68 (0.83, 3.11)	χ^2=0.019,
Women	446	1.75 (1.08, 2.73)	p=0.89
Other vascular disease			
Men	276	1.51 (0.72, 2.86)	χ^2=0.16,
Women	451	1.72 (1.06, 2.67)	p=0.69
All vascular disease			
Men	1611	1.45 (0.87, 2.31)	χ^2=0.044,
Women	1867	1.53 (1.08, 2.10)	p=0.83
Any kidney disease			
Men	828	0.90 (0.50, 1.51)	χ^2=0.015,
Women	1225	0.87 (0.59, 1.24)	p=0.90

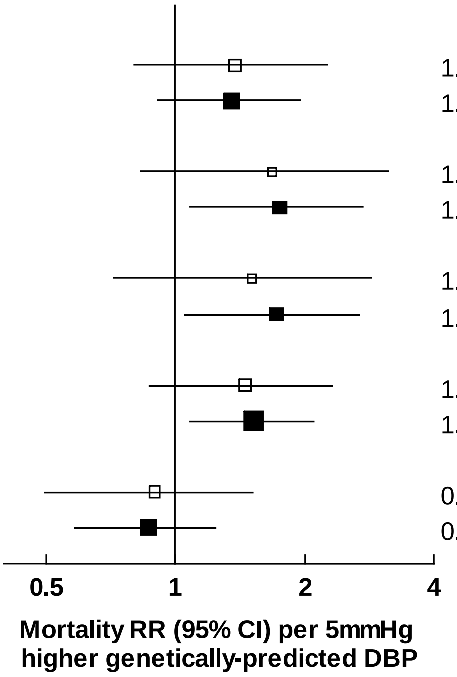

Mortality rate ratios (RRs) are per 5mmHg higher genetically-predicted DBP and are stratified by sex and adjusted for age-at-risk, body mass index, district of residence and the first 7 genetic principal components. The size of each square is inversely proportional to the variance of the log RR. Horizontal lines represent 95% confidence intervals (CI). DBP = diastolic blood pressure.

Figure S12: Associations between genetically-predicted SBP and cardiovascular and kidney mortality by district of residence

Cause of death	No. of deaths	RR (95% CI)	Heterogeneity test by district
Ischemic heart disease			
Coyoacán	682	1.92 (1.38, 2.65)	χ²=0.36,
Iztapalapa	1323	1.71 (1.30, 2.24)	p=0.55
Cerebrovascular disease			
Coyoacán	237	1.53 (0.93, 2.47)	χ²=1.7,
Iztapalapa	509	2.11 (1.44, 3.04)	p=0.19
Other vascular disease			
Coyoacán	254	1.68 (1.04, 2.66)	χ²=0.74,
Iztapalapa	473	1.34 (0.90, 1.96)	p=0.39
All vascular disease			
Coyoacán	1173	1.78 (1.34, 2.35)	χ²=0.075,
Iztapalapa	2305	1.70 (1.34, 2.15)	p=0.78
Any kidney disease			
Coyoacán	579	1.93 (1.35, 2.71)	χ²=4.1,
Iztapalapa	1474	1.26 (0.96, 1.63)	p=0.04

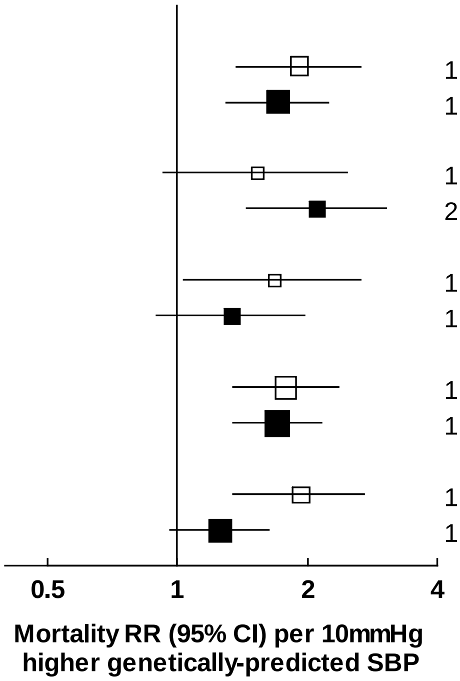

Mortality rate ratios (RRs) are per 10mmHg higher genetically-predicted SBP and are stratified by district of residence and adjusted for age-at-risk, sex, body mass index and the first 7 genetic principal components. The size of each square is inversely proportional to the variance of the log RR. Horizontal lines represent 95% confidence intervals (CI). SBP = systolic blood pressure.

Figure S13: Associations between genetically-predicted DBP and cardiovascular and kidney mortality by district of residence

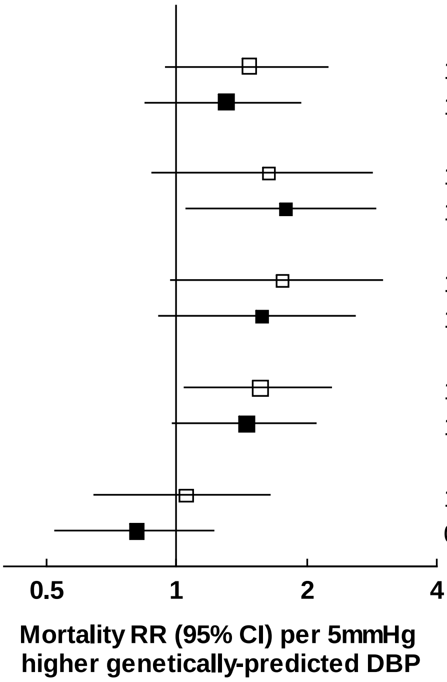

Cause of death	No. of deaths	RR (95% CI)	Heterogeneity test by district
Ischemic heart disease			
Coyoacán	682	1.48 (0.94, 2.23)	χ²=0.24,
Iztapalapa	1323	1.30 (0.85, 1.94)	p=0.62
Cerebrovascular disease			
Coyoacán	237	1.64 (0.88, 2.83)	χ²=0.081,
Iztapalapa	509	1.79 (1.05, 2.88)	p=0.78
Other vascular disease			
Coyoacán	254	1.76 (0.97, 2.99)	χ²=0.13,
Iztapalapa	473	1.57 (0.91, 2.58)	p=0.72
All vascular disease			
Coyoacán	1173	1.56 (1.05, 2.27)	χ²=0.11,
Iztapalapa	2305	1.45 (0.98, 2.09)	p=0.74
Any kidney disease			
Coyoacán	579	1.05 (0.65, 1.64)	χ²=0.91,
Iztapalapa	1474	0.81 (0.52, 1.22)	p=0.34

Mortality rate ratios (RRs) are per 5mmHg higher genetically-predicted DBP and are stratified by district of residence and adjusted for age-at-risk, sex, body mass index and the first 7 genetic principal components. The size of each square is inversely proportional to the variance of the log RR. Horizontal lines represent 95% confidence intervals (CI). DBP = diastolic blood pressure.

Figure S14: Associations between genetically-predicted SBP and cardiovascular and kidney mortality by ancestry group

Cause of death	No. of deaths	RR (95% CI)	Heterogenity test by ancestry
Ischemic heart disease			
Least IAM ancestry	676	1.89 (1.33, 2.66)	χ²=4.8,
Mid IAM ancestry	696	2.09 (1.48, 2.92)	p=0.09
Most IAM ancestry	633	1.34 (0.89, 1.98)	
Cerebrovascular disease			
Least IAM ancestry	268	1.73 (1.07, 2.76)	χ²=3.4,
Mid IAM ancestry	214	2.65 (1.57, 4.37)	p=0.19
Most IAM ancestry	264	1.52 (0.87, 2.58)	
Other vascular disease			
Least IAM ancestry	230	1.82 (1.09, 2.97)	χ²=2.6,
Mid IAM ancestry	244	1.46 (0.87, 2.37)	p=0.27
Most IAM ancestry	253	1.13 (0.63, 1.95)	
All vascular disease			
Least IAM ancestry	1174	1.84 (1.36, 2.47)	χ²=5.5,
Mid IAM ancestry	1154	2.03 (1.49, 2.72)	p=0.06
Most IAM ancestry	1150	1.33 (0.94, 1.85)	
Any kidney disease			
Least IAM ancestry	451	1.60 (1.07, 2.36)	χ²=0.55,
Mid IAM ancestry	646	1.39 (0.98, 1.96)	p=0.76
Most IAM ancestry	956	1.36 (0.95, 1.94)	

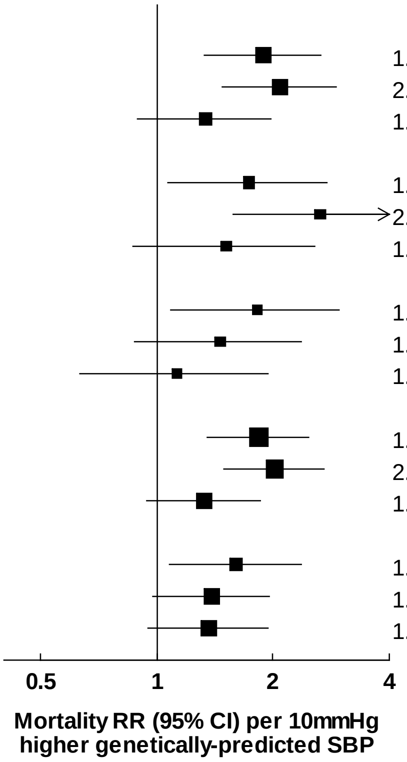

Mortality rate ratios (RRs) are per 10mmHg higher genetically-predicted SBP and are stratified by AMR ancestry and adjusted for age-at-risk, sex, body mass index, district of residence and the first 7 genetic principal components. The size of each square is inversely proportional to the variance of the log RR. Horizontal lines represent 95% confidence intervals (CI). IAM = Indigenous American, SBP = systolic blood pressure.

Figure S15: Associations between genetically-predicted DBP and cardiovascular and kidney mortality by ancestry group

Cause of death	No. of deaths	RR (95% CI)	Heterogeneity test by ancestry
Ischemic heart disease			
Least IAM ancestry	676	1.67 (1.05, 2.57)	χ^2=3.3,
Mid IAM ancestry	696	1.37 (0.81, 2.21)	p=0.19
Most IAM ancestry	633	0.98 (0.49, 1.79)	
Cerebrovascular disease			
Least IAM ancestry	268	1.20 (0.64, 2.10)	χ^2=5.2,
Mid IAM ancestry	214	2.59 (1.31, 4.72)	p=0.07
Most IAM ancestry	264	1.91 (0.85, 3.85)	
Other vascular disease			
Least IAM ancestry	230	1.77 (0.94, 3.09)	χ^2=0.37,
Mid IAM ancestry	244	1.44 (0.72, 2.67)	p=0.83
Most IAM ancestry	253	1.68 (0.73, 3.43)	
All vascular disease			
Least IAM ancestry	1174	1.57 (1.03, 2.32)	χ^2=0.62,
Mid IAM ancestry	1154	1.56 (0.97, 2.41)	p=0.73
Most IAM ancestry	1150	1.28 (0.70, 2.21)	
Any kidney disease			
Least IAM ancestry	451	1.07 (0.63, 1.74)	χ^2=1.1,
Mid IAM ancestry	646	0.89 (0.51, 1.47)	p=0.57
Most IAM ancestry	956	0.76 (0.40, 1.35)	

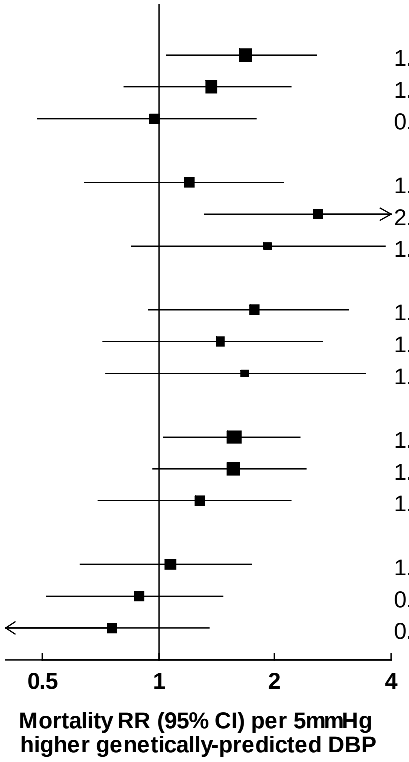

Mortality rate ratios (RRs) are per 5mmHg higher genetically-predicted DBP and are stratified by AMR ancestry and adjusted for age-at-risk, sex, body mass index, district of residence and the first 7 genetic principal components. The size of each square is inversely proportional to the variance of the log RR. Horizontal lines represent 95% confidence intervals (CI). DBP = diastolic blood pressure, IAM = Indigenous American.

Figure S16: Associations between genetically-predicted SBP and cause-specific mortality using AMR and trans-ancestry instruments

Cause of death	No. of deaths	RR (95% CI)
Ischemic heart disease		
AMR	2005	1.81 (1.42, 2.29)
Trans-ancestry	2005	1.78 (1.44, 2.19)
Cerebrovascular disease		
AMR	746	1.74 (1.24, 2.42)
Trans-ancestry	746	1.88 (1.40, 2.51)
Other vascular disease		
AMR	727	1.60 (1.13, 2.23)
Trans-ancestry	727	1.45 (1.07, 1.95)
All vascular disease		
AMR	3478	1.75 (1.42, 2.15)
Trans-ancestry	3478	1.73 (1.44, 2.06)
Chronic kidney disease		
AMR	1740	1.37 (1.06, 1.76)
Trans-ancestry	1740	1.45 (1.16, 1.80)
Other kidney disease		
AMR	313	1.23 (0.74, 1.99)
Trans-ancestry	313	1.29 (0.83, 1.98)
Any kidney disease		
AMR	2053	1.35 (1.06, 1.70)
Trans-ancestry	2053	1.42 (1.15, 1.75)
Acute diabetic crisis		
AMR	551	0.90 (0.61, 1.33)
Trans-ancestry	551	1.12 (0.79, 1.56)
Hepatobiliary disease		
AMR	1070	0.86 (0.63, 1.16)
Trans-ancestry	1070	0.88 (0.67, 1.14)
Infection		
AMR	816	1.06 (0.76, 1.47)
Trans-ancestry	816	1.02 (0.76, 1.36)
Cancer		
AMR	2116	1.00 (0.79, 1.26)
Trans-ancestry	2116	0.95 (0.77, 1.16)
Respiratory disease		
AMR	2072	0.97 (0.77, 1.23)
Trans-ancestry	2072	1.04 (0.84, 1.28)
External/other/ill-defined		
AMR	997	1.35 (0.99, 1.82)
Trans-ancestry	997	1.34 (1.02, 1.74)
All-cause mortality		
AMR	13 153	1.22 (1.04, 1.44)
Trans-ancestry	13 153	1.24 (1.08, 1.43)

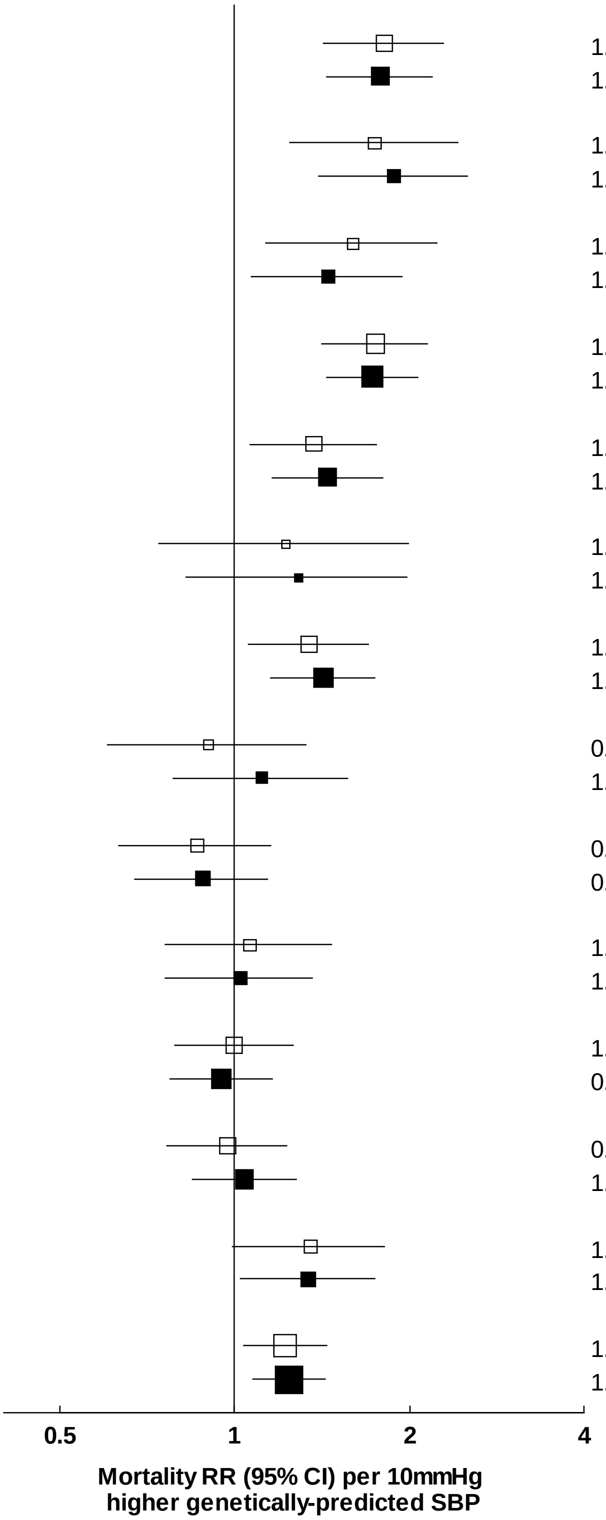

Mortality rate ratios (RRs) are per 10mmHg higher genetically-predicted SBP using genetic scores for SBP constructed with AMR and trans-ancestry weights. Both sets of analyses are adjusted for age-at-risk, sex, body mass index, district of residence and the first 7 genetic principal components. The size of each square is inversely proportional to the variance of the log RR. Horizontal lines represent 95% confidence intervals (CI). AMR: admixed American, SBP: systolic blood pressure.

Figure S17: Associations between genetically-predicted DBP and cause-specific mortality using AMR and trans-ancestry instruments

Cause of death	No. of deaths	RR (95% CI)
Ischemic heart disease		
AMR	2005	1.56 (1.15, 2.08)
Trans-ancestry	2005	1.36 (1.00, 1.82)
Cerebrovascular disease		
AMR	746	1.40 (0.94, 2.04)
Trans-ancestry	746	1.72 (1.16, 2.47)
Other vascular disease		
AMR	727	1.78 (1.20, 2.56)
Trans-ancestry	727	1.63 (1.10, 2.36)
All vascular disease		
AMR	3478	1.57 (1.19, 2.04)
Trans-ancestry	3478	1.48 (1.12, 1.93)
Chronic kidney disease		
AMR	1740	0.94 (0.68, 1.29)
Trans-ancestry	1740	0.85 (0.61, 1.17)
Other kidney disease		
AMR	313	1.11 (0.61, 1.88)
Trans-ancestry	313	1.02 (0.57, 1.74)
Any kidney disease		
AMR	2053	0.97 (0.70, 1.30)
Trans-ancestry	2053	0.88 (0.64, 1.18)
Acute diabetic crisis		
AMR	551	0.71 (0.43, 1.12)
Trans-ancestry	551	0.66 (0.40, 1.04)
Hepatobiliary disease		
AMR	1070	1.08 (0.74, 1.53)
Trans-ancestry	1070	1.27 (0.88, 1.79)
Infection		
AMR	816	0.96 (0.64, 1.41)
Trans-ancestry	816	1.02 (0.68, 1.48)
Cancer		
AMR	2116	1.10 (0.81, 1.47)
Trans-ancestry	2116	0.98 (0.72, 1.32)
Respiratory disease		
AMR	2072	1.01 (0.73, 1.35)
Trans-ancestry	2072	0.96 (0.70, 1.30)
External/other/ill-defined		
AMR	997	1.34 (0.92, 1.89)
Trans-ancestry	997	1.31 (0.90, 1.85)
All-cause mortality		
AMR	13 153	1.15 (0.90, 1.46)
Trans-ancestry	13 153	1.11 (0.86, 1.40)

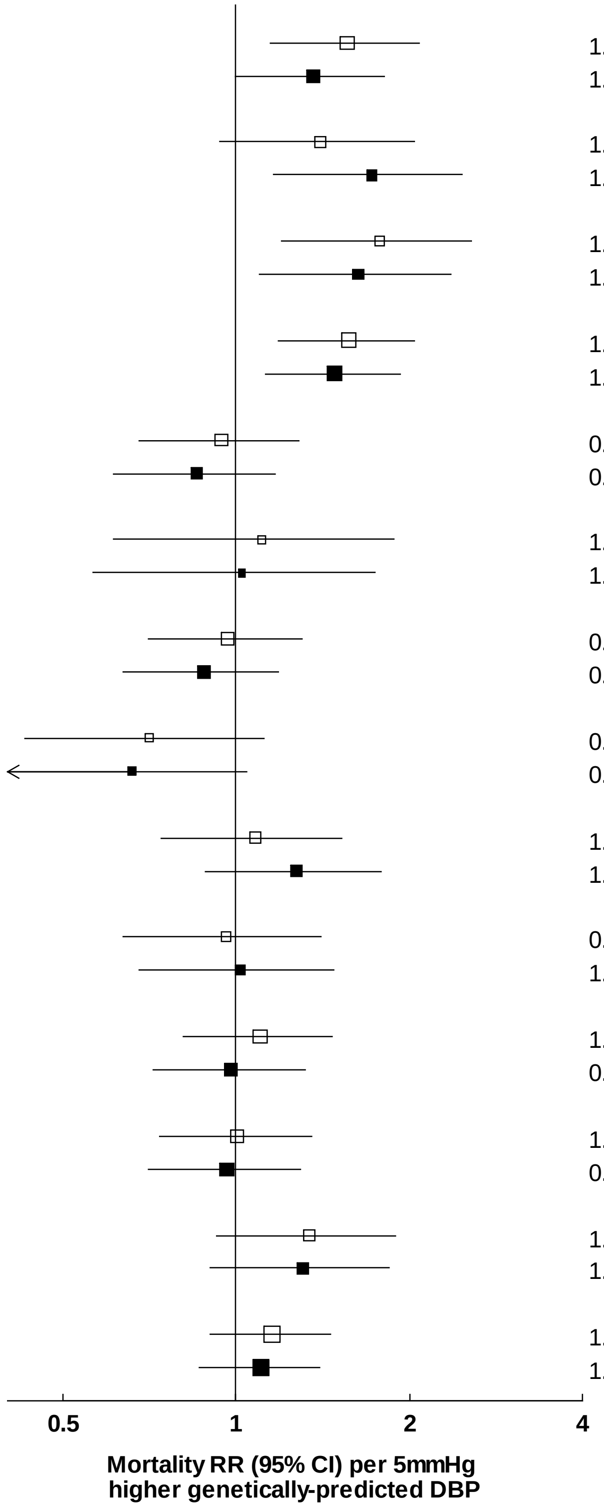

Mortality rate ratios (RRs) are per 5mmHg higher genetically-predicted DBP using genetic scores for SBP constructed with AMR and trans-ancestry weights. Both sets of analyses are adjusted for age-at-risk, sex, body mass index, district of residence and the first 7 genetic principal components. The size of each square is inversely proportional to the variance of the log RR. Horizontal lines represent 95% confidence intervals (CI). AMR: admixed American, DBP: diastolic blood pressure.

Figure S18: Associations between genetically-predicted SBP and cause-specific mortality in 72K unrelated participants and the whole cohort

Cause of death	No. of deaths	RR (95% CI)
Ischemic heart disease		
Unrelated	1095	1.68 (1.27, 2.19)
Whole cohort	2005	1.78 (1.44, 2.19)
Cerebrovascular disease		
Unrelated	422	1.72 (1.16, 2.51)
Whole cohort	746	1.88 (1.40, 2.51)
Other vascular disease		
Unrelated	412	1.98 (1.33, 2.88)
Whole cohort	727	1.45 (1.07, 1.95)
All vascular disease		
Unrelated	1929	1.74 (1.37, 2.20)
Whole cohort	3478	1.73 (1.44, 2.06)
Chronic kidney disease		
Unrelated	934	1.38 (1.02, 1.83)
Whole cohort	1740	1.45 (1.16, 1.80)
Other kidney disease		
Unrelated	178	1.09 (0.60, 1.90)
Whole cohort	313	1.29 (0.83, 1.98)
Any kidney disease		
Unrelated	1112	1.32 (1.00, 1.73)
Whole cohort	2053	1.42 (1.15, 1.75)
Acute diabetic crisis		
Unrelated	294	1.13 (0.71, 1.78)
Whole cohort	551	1.12 (0.79, 1.56)
Hepatobiliary disease		
Unrelated	530	0.85 (0.59, 1.22)
Whole cohort	1070	0.88 (0.67, 1.14)
Infection		
Unrelated	419	0.99 (0.66, 1.47)
Whole cohort	816	1.02 (0.76, 1.36)
Cancer		
Unrelated	1183	0.91 (0.69, 1.19)
Whole cohort	2116	0.95 (0.77, 1.16)
Respiratory disease		
Unrelated	1116	1.09 (0.83, 1.43)
Whole cohort	2072	1.04 (0.84, 1.28)
External/other/ill-defined		
Unrelated	542	1.44 (1.01, 2.03)
Whole cohort	997	1.34 (1.02, 1.74)
All-cause mortality		
Unrelated	7125	1.24 (1.03, 1.49)
Whole cohort	13153	1.24 (1.08, 1.43)

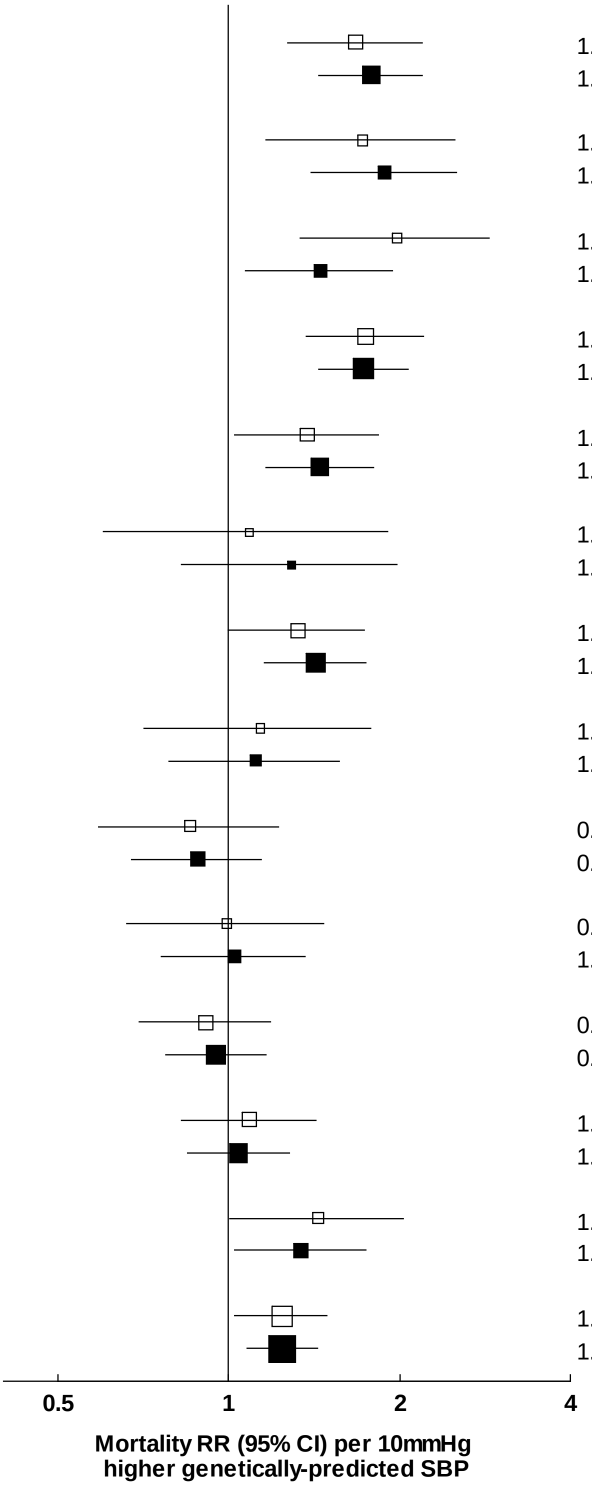

Mortality rate ratios (RRs) are per 10mmHg higher genetically-predicted SBP in analyses run in a set of 72K participants unrelated at the third degree, and the whole cohort. Both sets of analyses are adjusted for age-at-risk, sex, body mass index, district of residence and the first 7 genetic principal components. The size of each square is inversely proportional to the variance of the log RR. Horizontal lines represent 95% confidence intervals (CI). SBP: systolic blood pressure.

Figure S19: Associations between genetically-predicted DBP and cause-specific mortality in 72K unrelated participants and the whole cohort

Cause of death	No. of deaths	RR (95% CI)
Ischemic heart disease		
Unrelated	1095	1.43 (0.98, 2.05)
Whole cohort	2005	1.36 (1.00, 1.82)
Cerebrovascular disease		
Unrelated	422	1.55 (0.94, 2.44)
Whole cohort	746	1.72 (1.16, 2.47)
Other vascular disease		
Unrelated	412	1.59 (0.96, 2.51)
Whole cohort	727	1.63 (1.10, 2.36)
All vascular disease		
Unrelated	1929	1.49 (1.06, 2.06)
Whole cohort	3478	1.48 (1.12, 1.93)
Chronic kidney disease		
Unrelated	934	0.85 (0.55, 1.25)
Whole cohort	1740	0.85 (0.61, 1.17)
Other kidney disease		
Unrelated	178	0.97 (0.46, 1.88)
Whole cohort	313	1.02 (0.57, 1.74)
Any kidney disease		
Unrelated	1112	0.86 (0.58, 1.25)
Whole cohort	2053	0.88 (0.64, 1.18)
Acute diabetic crisis		
Unrelated	294	0.53 (0.27, 0.96)
Whole cohort	551	0.66 (0.40, 1.04)
Hepatobiliary disease		
Unrelated	530	1.13 (0.69, 1.76)
Whole cohort	1070	1.27 (0.88, 1.79)
Infection		
Unrelated	419	0.90 (0.53, 1.47)
Whole cohort	816	1.02 (0.68, 1.48)
Cancer		
Unrelated	1183	0.93 (0.63, 1.34)
Whole cohort	2116	0.98 (0.72, 1.32)
Respiratory disease		
Unrelated	1116	1.14 (0.77, 1.64)
Whole cohort	2072	0.96 (0.70, 1.30)
External/other/ill-defined		
Unrelated	542	1.48 (0.93, 2.26)
Whole cohort	997	1.31 (0.90, 1.85)
All-cause mortality		
Unrelated	7125	1.11 (0.82, 1.48)
Whole cohort	13153	1.11 (0.86, 1.40)

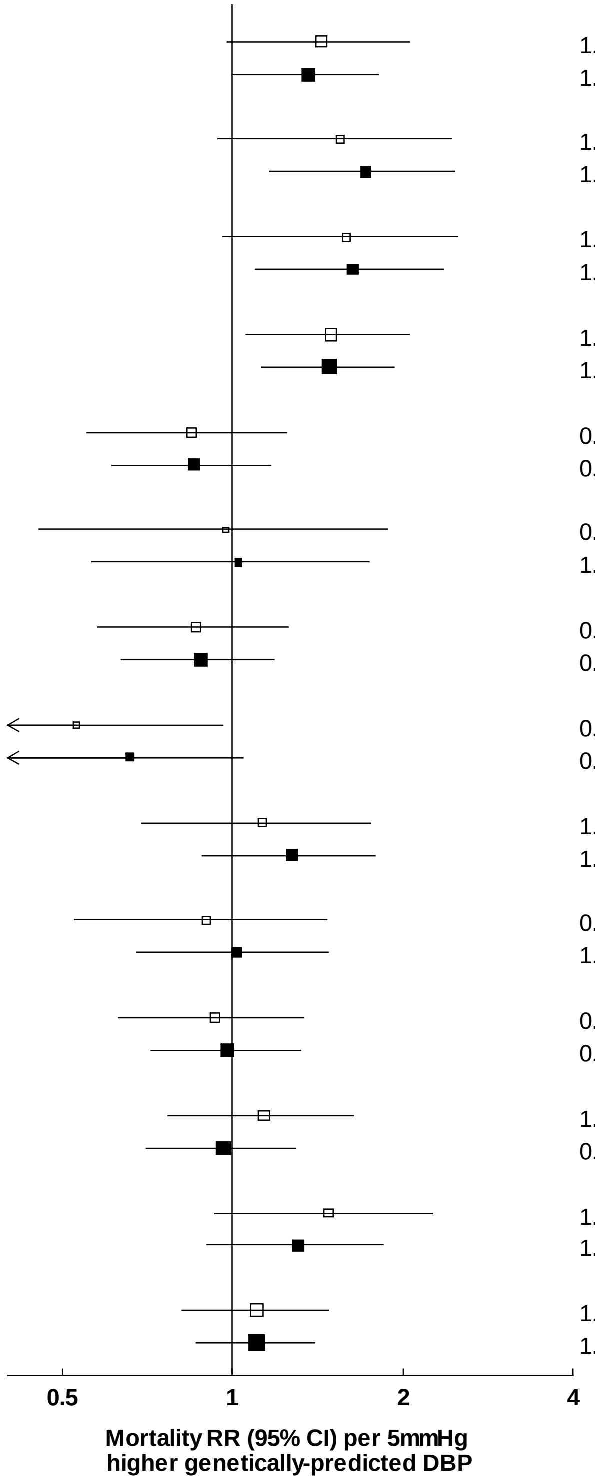

Mortality rate ratios (RRs) are per 5mmHg higher genetically-predicted DBP in analyses run in a set of 72K participants unrelated at the third degree, and the whole cohort. Both sets of analyses are adjusted for age-at-risk, sex, body mass index, district of residence and the first 7 genetic principal components. The size of each square is inversely proportional to the variance of the log RR. Horizontal lines represent 95% confidence intervals (CI). DBP: diastolic blood pressure.

Figure S20: Genetic associations of SBP and DBP with cause-specific mortality using a 15/10mmHg correction for antihypertensive use

Cause of death	A: Genetically-predicted SBP<br>No. of deaths	A: RR (95% CI)	B: Genetically-predicted DBP<br>No. of deaths	B: RR (95% CI)
**Vascular disease**				
Ischemic heart disease	2005	1.68 (1.39, 2.01)	2005	1.29 (1.01, 1.64)
Cerebrovascular disease	746	1.76 (1.35, 2.28)	746	1.58 (1.14, 2.14)
Other vascular disease	727	1.39 (1.06, 1.81)	727	1.51 (1.09, 2.06)
**Any vascular disease**	**3478**	**1.63 (1.39, 1.91)**	**3478**	**1.40 (1.12, 1.73)**
**Kidney disease**				
Chronic kidney disease	1740	1.39 (1.14, 1.69)	1740	0.88 (0.67, 1.13)
Other kidney disease	313	1.26 (0.84, 1.85)	313	1.02 (0.63, 1.59)
**Any kidney disease**	**2053**	**1.37 (1.14, 1.64)**	**2053**	**0.90 (0.70, 1.14)**
**Other causes**				
Acute diabetic crisis	551	1.10 (0.81, 1.49)	551	0.70 (0.47, 1.03)
Hepatobiliary disease	1070	0.89 (0.70, 1.13)	1070	1.23 (0.91, 1.62)
Infection*	816	1.02 (0.78, 1.32)	816	1.01 (0.73, 1.38)
Cancer	2116	0.96 (0.80, 1.14)	2116	0.98 (0.77, 1.25)
Respiratory disease**	2072	1.04 (0.86, 1.24)	2072	0.97 (0.75, 1.23)
External/other/ill-defined	997	1.30 (1.02, 1.64)	997	1.26 (0.93, 1.67)
**All-cause mortality**	**13153**	**1.21 (1.07, 1.37)**	**13153**	**1.09 (0.90, 1.31)**

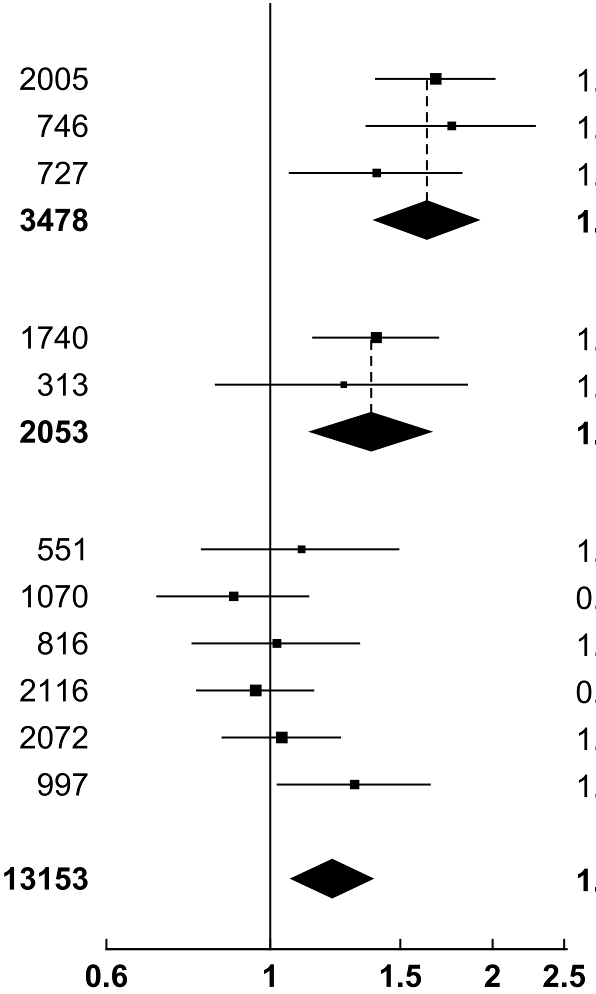

Mortality RR (95% CI) per 10mmHg higher genetically-predicted SBP

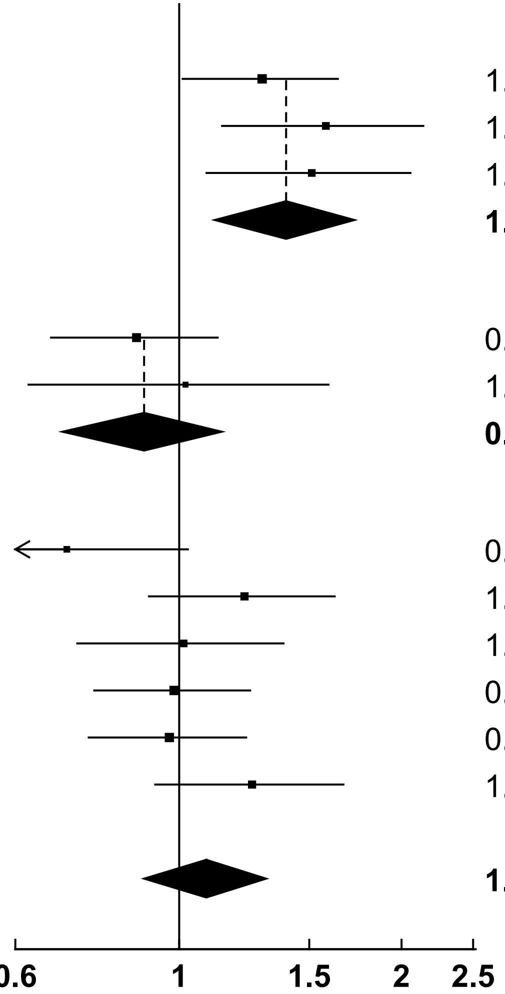

Mortality RR (95% CI) per 5mmHg higher genetically-predicted DBP

Mortality rate ratios (RRs) are per 10mmHg higher genetically-predicted SBP (A) or 5mmHg higher genetically-predicted DBP (B) when participants taking antihypertensive medications had their SBP increased by 15mmHg annd DBP by 10mmHg. Both sets of analyses are adjusted for age-at-risk, sex, body mass index, district of residence and the first 7 genetic principal components. The size of each square is inversely proportional to the variance of the log RR. Horizontal lines represent 95% confidence intervals (CI). *Excludes respiratory infections. **Includes respiratory infections. DBP: diastolic blood pressure, SBP: systolic blood pressure.
