# Supplementary material for: Blood Pressure and Mortality in Mexico City: A Mendelian Randomization Study
Source: Hypertension. 2025 Sep 17;82(11):1896–905. doi: 10.1161/HYPERTENSIONAHA.125.25348 (PMC7618184; doi:10.1161/HYPERTENSIONAHA.125.25348)
Supplement: Supplementary file 3 [file hyp-82-1896-s003.pdf]

*** Social Drivers of Health Checklist**
Does this study focus on research involving differences in sex, gender, race/ethnicity, or other demographic categories or any other topic that should be reviewed through a SDOH lens?
Yes

Disparities Research Reporting
Does this manuscript focus on reporting health differences by race and/or ethnicity? If so, a completed Disparities Research checklist [link] should be provided as a supplemental file.
No

Demographic Terminology
Does this manuscript adhere to the AMA's guidance for appropriate terminology for demographic information [PDF]? If historical terminology is used, is the usage explained?
Yes

Sex, Gender, and Race/Ethnicity Specific Results
Are sex, gender, and race/ethnicity specific results of the study's primary outcomes reported regardless of whether there are significant differences by sex or race? If not, the reason for not reporting these results should be provided in the manuscript.
Yes

Demographic Representation for Clinical Research
Were steps taken to ensure the study is demographically representative, and if not, is the focus on one group or exclusion of other groups scientifically and conceptually justified?
Yes

Date completed: 08/26/2025 09:10:02
User pid: 673850
